# Integrating RNA-seq and population genomics to elucidate salt tolerance mechanisms in flax (*Linum usitatissimum* L.)

**DOI:** 10.3389/fpls.2024.1442286

**Published:** 2024-11-19

**Authors:** Yuan-Dong Li, Xiao Li, Lei-Lei Zhu, Yang Yang, Dong-Liang Guo, Li-Qiong Xie

**Affiliations:** ^1^ Xinjiang Key Laboratory of Biological Resources and Genetic Engineering, College of Life Science and Technology, Xinjiang University, Urumqi, China; ^2^ Department of Basic Medicine, Xinjiang Second Medical College, Karamay, Xinjiang, China; ^3^ College of Smart Agriculture, Xinjiang University, Urumqi, China

**Keywords:** flax (*Linum usitatissimum* L.), salt stress, transcriptome sequencing, population level analysis, nucleotide diversity

## Abstract

Salinity is an important abiotic environmental stressor threatening agricultural productivity worldwide. Flax, an economically important crop, exhibits varying degrees of adaptability to salt stress among different cultivars. However, the specific molecular mechanisms underlying these differences in adaptation have remained unclear. The objective of this study was to identify candidate genes associated with salt tolerance in flax using RNA-Seq combined with population-level analysis. To begin with, three representative cultivars were selected from a population of 200 flax germplasm and assessed their physiological and transcriptomic responses to salt stress. The cultivar C121 exhibited superior osmoregulation, antioxidant capacity, and growth under salt stress compared to the other two cultivars. Through transcriptome sequencing, a total of 7,459 differentially expressed genes associated with salt stress were identified, which were mainly enriched in pathways related to response to toxic substances, metal ion transport, and phenylpropanoid biosynthesis. Furthermore, genotyping of the 7,459 differentially expressed genes and correlating them with the phenotypic data on survival rates under salt stress allowed the identification of 17 salt-related candidate genes. Notably, the nucleotide diversity of nine of the candidate genes was significantly higher in the oil flax subgroup than in the fiber flax subgroup. These results enhance the fundamental understanding of salt tolerance mechanisms in flax, provide a basis for a more in-depth exploration of its adaptive responses to salt stress, and facilitate the scientific selection and breeding of salt-tolerant varieties.

## 1 Introduction

Soil salinity is a significant abiotic stress that primarily occurs in coastal and arid/semi-arid regions, greatly impacting plant life processes and ultimately limiting agricultural productivity and crop worldwide distribution (Chen et al., 2023; Munns and Gilliham, 2015). Currently, more than 20% of cultivated land (equivalent to 1000 million hectares) is impacted by salt, a situation that is worsening due to global climate change and insufficient management of irrigation and fertilizer practices (Corwin, 2020; Jiang et al., 2019). The most effective approach to mitigate yield losses in plant production is the development of plant varieties that are both salt-tolerant and high-yielding (van Zelm et al., 2020). Comprehending the mechanisms underlying plants’ response to salinity is crucial for implementing traditional breeding techniques and biotechnological approaches to enhance stress resistance in plants (Ashraf et al., 2013).

High salinity poses multiple challenges to plants, including hyperosmotic stress, oxidative stress, ionic imbalance, and metabolic disorders (Li et al., 2022b). Plants have evolved a variety of morphological, physiological, biochemical, and molecular adaptation strategies to withstand salt stress and sustain growth, development, and productivity (Arif et al., 2020). Deeper and more comprehensive mechanisms underlying plant responses to salt stress have resulted from research conducted over the past two decades in many crops, which has increasingly relied on genetic and biochemical analyses (Atta et al., 2023; Yang and Guo, 2018). Many biological pathways and genes that respond to salt have been found in a wide range of species, and they have roles in ion accumulation and exclusion, transcription control, stress signal transmission, redox processes, and the accumulation of certain osmoregulation chemicals. Some examples of these genes include transcription factor genes such as *OsDOF15* (Qin et al., 2019), *OsbHLH38* (Du et al., 2023), and *GsMYB15* (Shen et al., 2018), kinase genes like *OsMKK10.2* (Yu et al., 2023), *STRK1* (Zhou et al., 2018), and *GmSNF1* (Lu et al., 2023), metabolism-related genes such as *GmNmrA6* (Mao et al., 2023), and DNA demethylase genes such as *OsDML4* (Li et al., 2023). It is noteworthy that modifying AT1 or its orthologs in monocot crops has shown great promise in improving saline-alkaline tolerance in sorghum, rice, and maize (Zhang et al., 2023). Though new genes for salt response were reported in recent years, related research in non-model and minor crops still lacking. The structural diversity and evolution of complexity in crops make it necessary to screen and dig for new genes in more crops to detail the strategies for salt stress.

Flax (*Linum usitatissimum* L.) is one of the oldest crops that has garnered increased attention due to its remarkable health benefits and rich nutrient content (Hall et al., 2016). Currently, there is an urgent need for salt-tolerant flax varieties in salt-affected zones, particularly in China’s Xinjiang and Inner Mongolia regions, as well as several countries in the Middle East that lack irrigating water and suffer from hazards of saline soils (Abido and Zsombik, 2019; Li et al., 2022a). Some studies have focused on physiological and biochemical responses to salt stress (Abido and Zsombik, 2019; Mekawy et al., 2019) and transcriptome analysis of individual materials (Wang et al., 2022; Wu et al., 2019; Yu et al., 2014) in linseed, providing theoretical support for our understanding of flax salt responses. However, less recognized are the adaptations in flax root morphology and their relevance for salt tolerance, as well as the variations in salt tolerance observed among different cultivars.

In this study, we systematically analyzed the growth parameters, antioxidant capacity, water-holding capacity, and root transcriptomic profile of three flax cultivars. The results demonstrated that a powerful antioxidant system and osmoregulatory capacity were important contributors to the flax’s resistance to salt stress. Meanwhile, we genotyped the differentially expressed genes for salt stress in the three materials and further screened the salt-related candidate genes by combining the phenotypic data of 200 flax populations. This study aims to identify candidate genes related to salt tolerance, providing a foundation for a better understanding of the molecular processes involved in salt stress response and for improving the resistance of flax.

## 2 Materials and methods

### 2.1 Plant materials and treatment

A Genome-wide Association Study (GWAS) population containing 200 diverse flax accessions was previously established (Guo et al., 2020), and all cultivars within this population were evaluated for salt tolerance during germination (Li et al., 2022) and seedling stages (**Supplementary Figure S1**; **Supplementary Table S1**). Interestingly, we observed that some cultivars exhibited inconsistent salt tolerance between the germination and seedling stages. To gain insight into the salt tolerance traits of flax, we utilized seedling stage results as a reference point to choose one salt-tolerant cultivar, C121, along with two salt-sensitive cultivars, C71 and C49 (demonstrating salt tolerance during germination), for experimental analysis. The seedling planting methods were described previously (Li et al., 2022a). Briefly, 36 seeds of each cultivar were planted in 96-well PCR plates and three PCR plates were incubated in a hydroponic tank for 21 days. Then the group treated with 200 mmol/L NaCl was labeled as “TR” (treated) group and the group without salt solution was labeled as “CK” (control) group. Each hydroponic tank was designated as one biological replicate, and the experiment was repeated at least three times to ensure accurate data.

### 2.2 Statistics on phenotypic traits

In the hydroponic experiment, 36 seedlings of each flax cultivar were planted together in the same hydroponic pot, which represented one replicate. The plants were exposed to salt stress for 7 days. After this period, the surviving plants were manually counted, and photographs were taken. To further analyze the effects of salt stress on the flax seedling root systems, a random sample of six seedlings was taken from each replicate. The aerial part of the seedlings was removed, and the remaining root portion was analyzed using the LA-S series plant root analysis system (Wan Shen, Hangzhou, China). 9 plants were randomly selected from each sample for measuring the dry and fresh weights of the whole plant, which were then used to calculate the relative water content as described previously (Li et al., 2022a). Relative survival refers to the ratio of green seedlings before and after stress, whereas relative root change is calculated as the difference between stressed root data and pre-stress root data, divided by the mean pre-stress root value.

Due to the complexity of the GWAS population, we implemented another program to count survival rates. The planting and salt stress treatment methods for the flax seedlings were as follows. Initially, 40 seeds from each flax cultivar were selected and placed in seedling pots containing a mixture of perlite, vermiculite, and nutrient soil in a ratio of 1:1:2. After a germination period of 14 days, the seedling pots were transferred to a hydroponic tank for salt stress treatment. The treatment solution was refreshed every 7 days. The seedling survival rate was measured after 21 days of salt stress and then combined with the pre-stress survival rate to determine the relative survival rate. The results from multiple experiments confirmed 450 mmol/L NaCl as the optimal salt stress concentration (**Supplementary Figure S1A**). In this experiment, we conducted three biological replicates and the correlation coefficient was greater than 0.6 (**Supplementary Figures S1B, C**). Varieties with relative survival rates exceeding 0.5 in all three experiments were classified as salt-tolerant, whereas those with rates below 0.05 were categorized as salt-sensitive materials (**Supplementary Table S1**).

### 2.3 Determination of physiological and biochemical indicators

The Nanjing Jiancheng Bioengineering Institute (http://www.njjcbio.com/) provided kits for measuring the following physiological indicators: soluble sugar content, proline content, catalase (CAT) enzyme activity, and malondialdehyde (MDA) content. Specifically, the Plant Soluble Sugar Content Test Kit (A145-1-1) was used to determine soluble sugar levels, employing the anthrone colorimetric method. The Proline Assay Kit (A107-1-1) relied on the ninhydrin reaction to assess proline content. For CAT activity, the Catalase Assay Kit (A007-1-1) utilized the ammonium molybdate method, which required an initial determination of protein concentration in the supernatant using Thomas Brilliant Blue. CAT activity was then expressed as units per milligram of protein, with one unit defined as the consumption of 1 µmol of H_2_O_2_ by 1 mg of tissue protein at 405 nm in 1 second. Finally, the Plant Malondialdehyde Kit (A003-3-1) measured MDA content using the thiobarbituric acid method. After 3 days of salt stress, the relevant osmoregulatory substances and antioxidant enzyme activities of flax reached a peak. 0.1g of seedling root from 3 days of treatment was required for each experiment. The corresponding indexes were tested according to the kit instructions.

Tetranitroblue tetrazolium chloride (NBT) staining was used to detect O_2_^• −^ content and diaminobenzidine (DAB) staining was used to detect H_2_O_2_ content, following previously published protocols (Yu et al., 2019). Seedling leaves from 3 days of treatment were employed in the experiments. The Soil and plant analyzer develotrnent (SPAD) values were measured using a SPAD-502 chlorophyll meter on the latest fully expanded leaf of each seedling. Eighteen seedlings were randomly selected from each sample, and the leaves from 3 days of treatment were used for the experiments.

### 2.4 RNA extraction, cDNA library construction and sequencing

Fresh roots from 3 days of treatment were collected in liquid nitrogen for total RNA extraction, the procedure was carried out according to the instructions of the RNeasy Plant Mini Kit (3 biological replicates each of the control and salt stress treatments, each replicate consisted of 3 plant roots). RNA purity and integrity were analyzed using agarose gel electrophoresis and further detected using Nanodrop and Agilent 2100, while RNA concentration was accurately quantified using Qubit 2.0. Subsequently, cDNA libraries were constructed and sequenced at Novogene (Beijing, China).

### 2.5 Sequencing data processing and identification of DEGs

After obtaining the sequencing data and checking the QC report, the next step was to process the data. Firstly, the flax reference genome and annotation file (2014 version) were downloaded from Phytozome (https://phytozome-next.jgi.doe.gov/). Using hisat2 v2.1.0, an index was built to compare the unzipped clean reads with the reference genome. Next, featurecounts v1.6.0 were used to count the read numbers mapped to each gene (Liao et al., 2014). Then, the transcripts per kilobase of million mapped reads (TPM) for each gene were calculated based on the length of the gene and the read count mapped to this gene. The limma program was used to correct the results obtained in the previous step and obtain the TPM and trimmed mean of M values (TMM) two-fold normalized matrices for gene expression data (Wagner et al., 2012).

Differential expression analysis was performed using the DESeq2 R package (1.20.0) (Love et al., 2014). The *p*-values were adjusted using the Benjamini–Hochberg method to control the false discovery rate. A corrected *p*-value of 0.05 and log_2_ (fold change) of 1 was set as the threshold for significance differential expression.

### 2.6 GO and KEGG enrichment analysis of DEGs

As described previously (Li et al., 2022a), the differentially expressed genes (DEGs) were enriched by GO and KEGG pathways using ClusterProfile R packages. Set *p*-value ≤ 0.05 as the standard to screen the path of GO and KEGG enrichment pathways.

### 2.7 Validation of DEGs by qRT-PCR

Six genes, namely *Lus10002916*, *Lus10020718*, *Lus10013250*, *Lus10012145*, *Lus10005114*, and *Lus10027742*, were randomly selected and verified by qRT-PCR using L*. usitatissimum ACT1* (GenBank accession number AY857865) as an internal reference gene in this experiment. The RNA samples used were consistent with the sequencing samples. Primer sets were designed using Primer Premier v6.25 and are listed in **Supplementary Table S2**. qRT-PCR was performed using the SYBR Green Master Mix kit, and the gene expression was detected using an ABI PRISM 7500 Real-time PCR system (USA). The following cycling conditions were used: 95°C for 3 min; 95°C for 15 s, 60°C for 60 s, and 40 cycles. The data were analyzed using the 2^−ΔΔCt^ method, and all samples were tested in triplicate.

### 2.8 Population-level screening

In total, 7,459 salt-responsive genes were mapped into a high-density flax genomic variation map from our previous study (Guo et al., 2020), and a total of 42649 SNPs were obtained by VCFtools v0.1.16. A population-level screening was performed using the GLM and MLM with TASSEL 5.0 (Bradbury et al., 2007). For GLM analysis, the population structure (Q) matrix generated from Admixture 1.23 was used to adjust for population stratification (GLM-Q). The kinship matrix (K) was calculated using TASSEL 5.0. The population structure matrix and Kinship matrix both were considered in the MLM (Q + K). To set the threshold cutoff level, we screened valid SNP loci by KGGSEE v1.0 (Li et al., 2011). The effective number of independent tests for all the 3,171 valid variants was 2,015.14 (63.55%). The *p*-value cutoff by Bonferroni correction for family-wise error rate 0.05 was 2.48e-05. The allele frequency and nucleotide diversity (*π*) were calculated in the software VCFtools (Li et al., 2020). The *π* values were analyzed utilizing a window size of 100 bp and a step size of 25 bp.

### 2.9 Identification of transcription factor gene family

Using the hidden Markov model (HMM), we obtained sequence-based transcription factor families. First, we downloaded the domain files corresponding to MYB (PF00249), bHLH (PF00010), and WD40 (PF00400) from the Pfam database (http://pfam.xfam.org/). Then, we compared the HMM files with the whole genome protein sequences of flax using hmmer 3.0 to identify proteins containing relevant domains. These proteins were further conditionally screened based on E-value ≤ 1e-5. Subsequently, we extracted the filtered protein sequences using Seqtk 1.3 (https://github.com/lh3/seqtk). The extracted protein sequences were then uploaded to the MEME database (https://meme-suite.org/meme/tools/meme) for motif prediction. Finally, the identified target proteins were annotated and validated using the Arabidopsis database (https://www.arabidopsis.org/), eggNOG-mapper (http://eggnog-mapper.embl.de/), and phytozome database.

### 2.10 Phylogenetic analysis

For efficient and accurate identification of homologous genes, it is crucial to establish a local blast database. To accomplish this, we obtained genome protein sequences of relevant species from the Ensembl Plants database (http://plants.ensembl.org/index.html) and integrated them with flax genome protein sequences into the local blast database. Subsequently, blastp analysis was conducted between the protein sequences of the target genes and the local database, using two key parameters (% Identity > 50 and E-value < 1e-30) for homologous protein screening. To further analyze the obtained similar sequences, we employed Seqtk 1.3 to swiftly extract them, followed by the construction of a phylogenetic tree. The complete amino acid sequence was aligned using ClustalW, and the phylogenetic tree was constructed by the neighbor-joining method with 1000 bootstrap replicates in MEGA10 software (https://www.megasoftware.net/).

### 2.11 Statistical analysis

All the experiments were repeated three times. The experimental data processing and mapping were completed using Excel 2020, GraphPad Prism 9.5, R version 4.0.4, IBM SPSS statistics 20.0, and TBtools-II. All values are expressed as mean ± standard error. The mean was compared using analysis of variance (ANOVA) and Duncan’s honestly significant difference test or independent samples *t*-test. The significance of data analyzed by independent samples *t*-test is denoted by an asterisk (*P* < 0.05), while significance according to Duncan’s test is indicated by different letters.

## 3 Results

### 3.1 Phenotypic and physiological evaluation for salinity stress response

**Figure 1:**
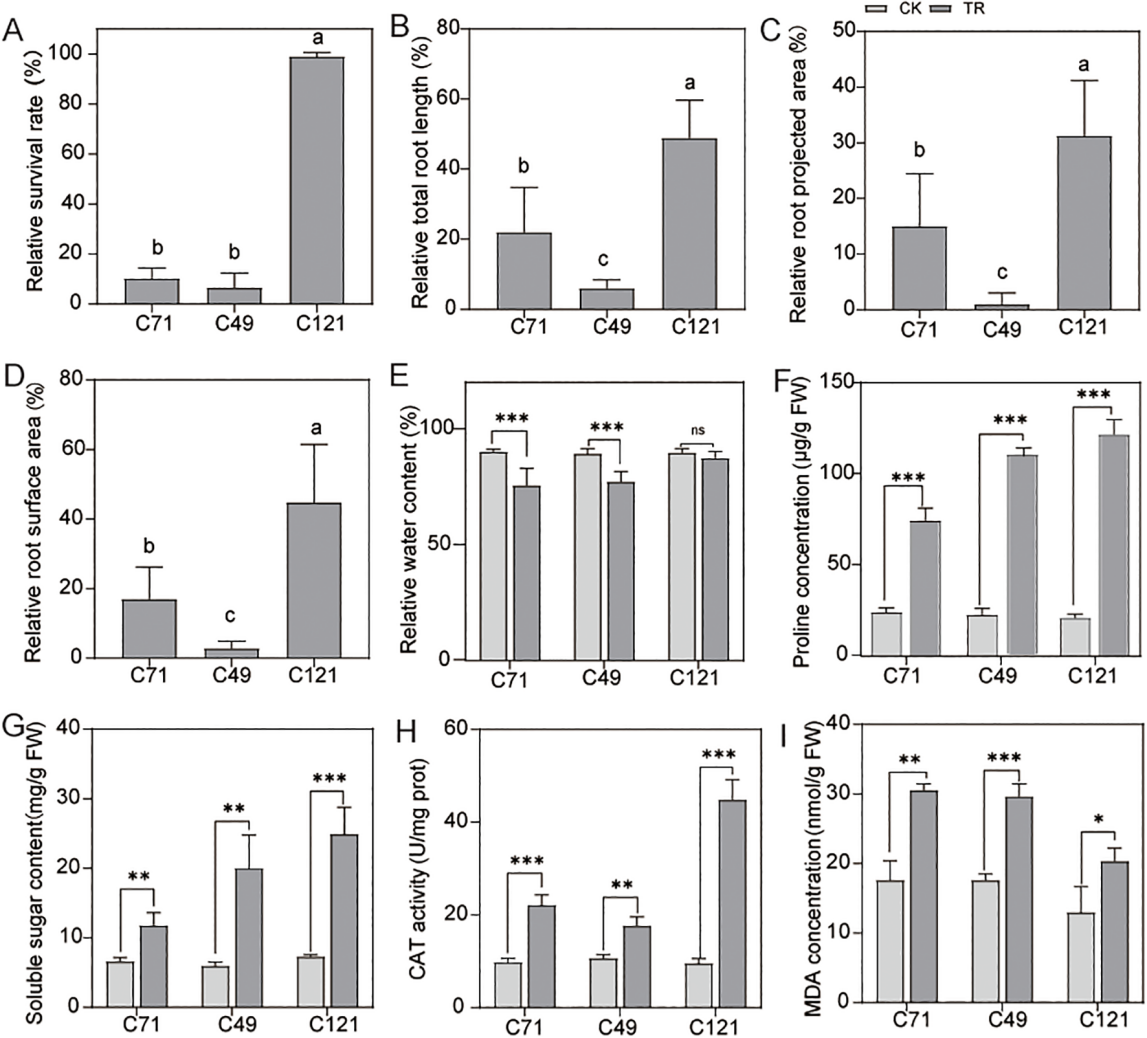
Detection of growth and physiological indices of flax under normal conditions and salt treatment. Phenotypes of the three cultivars were counted for **(A)** relative survival, **(B)** relative total root length, **(C)** relative root surface area, **(D)** root projected area, and **(E)** relative water content; **(F–I)** Effects of salt stress on the physiological parameters (contents of soluble sugar, proline and MDA, the activities of CAT) of roots in C49, C71 and C121. Values are means standard deviations (n = 3). The bars represent standard error. Different letters indicate significant differences based on Duncan’s test (*P* < 0.05). Independent samples *t*-test, ns without significant differences, *P < 0.05, **P < 0.01, ***P < 0.001. CK: 0 mmol/L NaCl, TR: 200 mmol/L NaCl.

Our results indicated that flax of different genotypes has shown varying degrees of salt adaptation (**Supplementary Table S1**), which of them with the accession of C121 exhibited a higher survival rate under 200 mmol/L NaCl stress, evidencing its salt-tolerant properties. In contrast, the other two flax cultivars C49 and C71 were found to be salt-sensitive (**Figure 1A**; **Supplementary Figure S2A**). Under normal conditions, C121 exhibited significantly slower growth rates in plant shoots and roots compared to the two salt-sensitive varieties (**Supplementary Figures S2B, C**). However, after 7 days of exposure to a salt solution, C121 showed remarkable root growth, whereas the root parameters of the salt-sensitive variety C49 remained almost unchanged (**Figures 1B–D**). Total root length, root projected area, and root surface area were increased by 22.01%, 15.02%, and 17.03% in cultivar C71, 5.96%, 1.07%, and 2.96% in cultivar C49, and 48.91%, 31.23% and 44.78% in cultivar C121, respectively.

Compared to the control group, the water content of C49, C71, and C121 decreased by an average of 13.51%, 16.34%, and 2.52%, respectively, under 200 mmol/L NaCl stress (**Figure 1E**). Interestingly, the concentrations of soluble sugars and proline were significantly higher in C49 and C121 than in C71 under 200 mmol/L NaCl stress in the roots, although no difference was observed under normal conditions (**Figures 1F, G**). Further, we conducted reactive oxygen species-related tests on these cultivars. NBT and DAB staining revealed that leaves treated with salt stress showed more intense staining in C49 and C71 compared to C121, indicating that C121 accumulated fewer O_2_^• −^ and H_2_O_2_ under salt stress (**Supplementary Figures S2D, E**). Consistently, when compared to the control condition, the activity of the antioxidant enzyme CAT increased by an average of 0.66-fold, 1.26-fold, and 3.69-fold in C49, C71, and C121, respectively, under 200 mmol/L NaCl stress (**Figure 1H**). MDA, a marker of lipid peroxidation and stress resistance (El Mamoun et al., 2023), was found to be significantly higher in C49 and C71 compared to C121 under 200 mmol/L NaCl stress, but there was no difference under normal conditions (**Figure 1I**). It is worth noting that SPAD value, which correlates with plant photosynthesis and can be used to measure plant growth rate (Jiang et al., 2017), also showed different trends among the leaves of three cultivars under salt stress, according to our results (**Supplementary Figure S2F**). Overall, our results demonstrate that C121 is capable of maintaining growth and displaying improved water retention and antioxidant capacity under salt stress, significantly outperforming the two sensitive varieties.

### 3.2 General transcriptome feature

**Figure 2:**
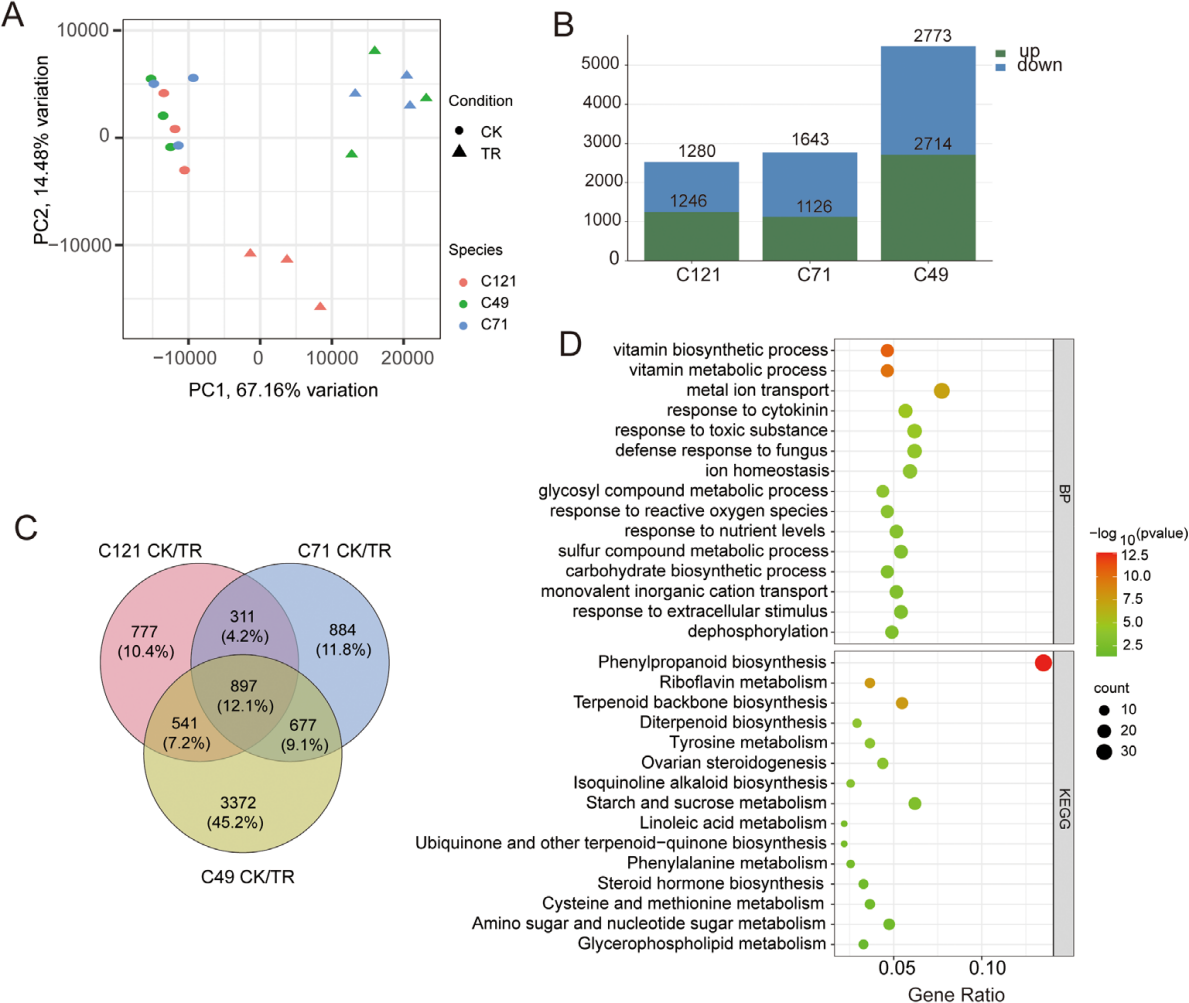
Counting and analysis of DEGs in flax under salt stress. **(A)** PCA analysis of correlations in 18 samples; **(B)** statistics of DEGs in three varieties under salt stress; **(C)** Venn diagrams of DEGs under salt stress from three cultivars and **(D)** bubble plots based on TOP15 terms from GO and KEGG enrichment.

To explore the adaptive response mechanisms of the three cultivars under salt stress, we conducted transcriptome sequencing of flax root systems before and after salt stress. Three biological replicates were performed for each cultivar. Mapping the rRNA depleted 727.1 million RNA-seq reads of the 18 samples against the flax reference genome (https://phytozome-next.jgi.doe.gov/info/Lusitatissimum_v1_0) showed that 650.6 million reads (91.1%) were mapped in total, and 623.1 million (87.2%) were mapped uniquely (**Supplementary Table S3**). The data from 43,471 gene expressions were obtained, and Spearman correlation coefficients of duplicate samples were always higher than 0.9 (**Supplementary Figure S3**). PCA analysis showed that salt stress affected gene expression in flax, and the effects differed somewhat between salt-tolerant and salt-sensitive samples (**Figure 2A**), with PC1 and PC2 explaining a total of 81.64% of the total variation in gene expression. To make the RNA data more convincing, we randomly selected six genes for qPCR validation. Combining RNA-seq and qRT-PCR data to calculate their correlation, the result showed a positive correlation with the Pearson coefficient *R* ^2^ = 0.944 (**Supplementary Figure S4**).

### 3.3 Insights into the critical biological processes under salt stress

Using DESeq2 to compare the differences between the three cultivars before and after salt treatment, we obtained a total of 7,459 differentially expressed genes (DEGs) related to salt response (**Supplementary Table S4**). These DEGs included 2,526 in C121 (1246 upregulated and 1280 downregulated), 2,769 in C71 (1,126 upregulated and 1,643 downregulated), and 5,487 in C49 (2,714 upregulated and 2,773 downregulated) (**Figure 2B**; **Supplementary Figures S5A–C**). GO and KEGG enrichment analyses of the DEGs in the three flax varieties revealed significant enrichment in response to toxic substances, metal ion transport, phenylpropanoid biosynthesis pathway, and more (**Supplementary Figures S5D–I**). These are common primarily metabolic pathways that plants activate in response to abiotic defense.

Furthermore, we identified a total of 899 common differentially expressed genes (DEGs) by merging the salt-responsive DEGs from the three flax cultivars (**Figure 2C**), which are considered to be the core regulatory genes for salt induction in flax. These DEGs were significantly enriched in 95 biological processes, 27 molecular functions, and 4 cellular compartments. Notably, there were 30 DEGs associated with metal ion transport, 24 with response to toxic substances, 23 with ion homeostasis, 22 with response to cytokinins, and 20 with response to nutrient levels. Additionally, the KEGG pathway enrichment analysis revealed significant enrichment of these DEGs in 21 pathways. Specifically, we found 37 DEGs enriched in phenylpropanoid biosynthesis, 17 in starch and sucrose metabolism, 15 in terpenoid backbone biosynthesis, 13 in amino sugar and nucleotide sugar metabolism, 10 in riboflavin metabolism, and 9 in steroid hormone biosynthesis (**Figure 2D**; **Supplementary Table S5**).

This suggests that flax plants employ various mechanisms, including enhanced energy utilization through starch and sucrose metabolism, to facilitate ion transport and detoxification reactions under salt stress conditions (**Supplementary Figures S5G–I**). Notably, a significant enrichment of common DEGs in the dephosphorylation process was observed, which is consistent with the energy dynamics and cellular signaling events that occur during stress response (**Figure 2D**).

### 3.4 Differences in transcripts of salt-tolerant and salt-sensitive varieties exposed to salt stress

**Figure 3:**
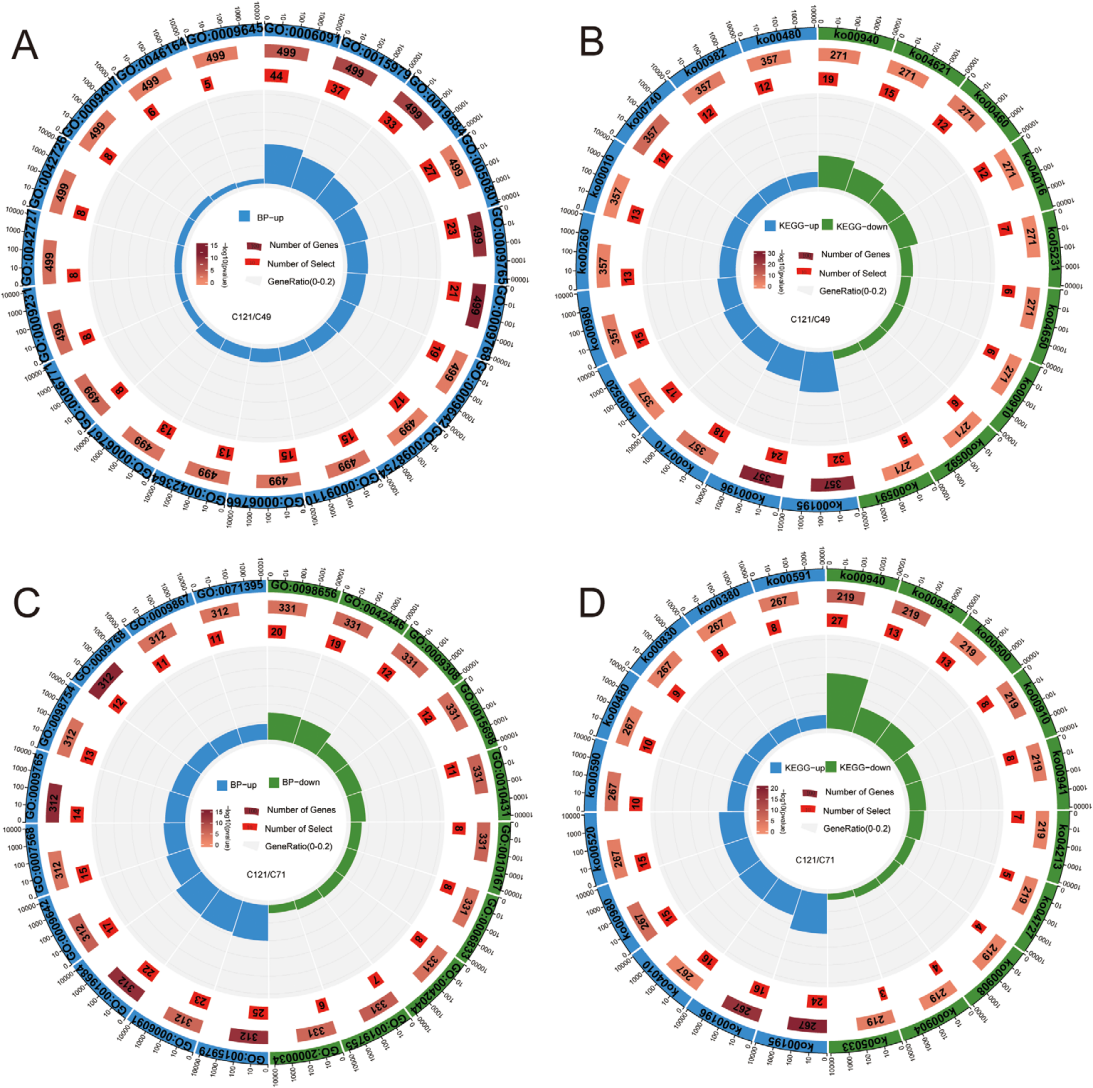
GO and KEGG enrichment circle maps based on salt‐tolerant and salt-sensitive cultivars of flax. Analysis of GO enrichment **(A)** and KEGG enrichment **(B)** based on the DEGs of C121/49 during salt stress; GO enrichment **(C)** and KEGG enrichment **(D)** based on the DEGs of C121/71 during salt stress. From outer to inner enrichment circle plots show: total number of genes in the enrichment pathway set (de-duplicated), annotation terms, annotated total number of DEGs (color represents significant), enriched number of DEGs in term, enriched gene percentage (enriched DEGs/annotated total DEGs), and detailed description. Terms information was presented in **Supplementary Table S6**.

Comparing the transcript differences between C121 and C49, 1852 DEGs (957 upregulated and 895 downregulated) were obtained under normal conditions, and 1,397 DEGs (800 upregulated and 597 downregulated) under salt stress (**Supplementary Figures S6A, B**). GO and KEGG enrichment analyses were used to determine the biological functions and pathways that underlie the 1,093 DEGs that were upregulated and 951 DEGs that were downregulated in C49 during salt stress as compared to C121 (**Figures 3A, B**; **Supplementary Table S6**). The results showed that the upregulated DEGs primarily correlated with processes such as precursor metabolite and energy generation, photosynthesis, ion homeostasis, detoxification, water-soluble vitamin biosynthesis, riboflavin metabolism, glutathione metabolism, etc.

Similarly, we compared the transcriptional differences between C121 and C71 during salt stress conditions, and the analysis indicated that 834 DEGs were upregulated and 709 DEGs were downregulated in C71 compared to C121 (**Supplementary Figures S6A, C**). The GO and KEGG enrichment demonstrated that the upregulated genes were predominantly enriched in aging, detoxification, jasmonic acid-mediated signaling pathways, MAPK signaling pathway, amino sugar, and nucleotide sugar metabolism, among others (**Figures 3C, D**; **Supplementary Table S6**). Interestingly, both analyses showed that C121 exhibited a greater number of upregulated genes related to the secondary metabolic pathways compared to the two salt-sensitive varieties, such as stilbenoid, diarylheptanoid, and gingerol biosynthesis, phenylpropanoid biosynthesis, and sphingolipid signaling pathway.

Furthermore, we aggregated the DEGs of the two groups and obtained 497 overlapping genes (287 downregulated DEGs and 210 upregulated DEGs compared to the two salt-sensitive varieties) (**Supplementary Figure S6D**). These genes were then aligned with Arabidopsis and predicted using the String online protein-protein interaction network (https://cn.string-db.org/). Interestingly, we haven’t only identified a strongly correlated regulatory network associated with photosynthesis, but also identified important salt-tolerant regulatory proteins in upregulated DEGs, such as CBL4, CBL8, MYB121, PP2CA, PIP2:2, SNRK2E, CIPK10, and so on (**Supplementary Figures S6E, F**; **Supplementary Table S7**).

### 3.5 DEGs involved in glutathione metabolism and riboflavin metabolism

**Figure 4:**
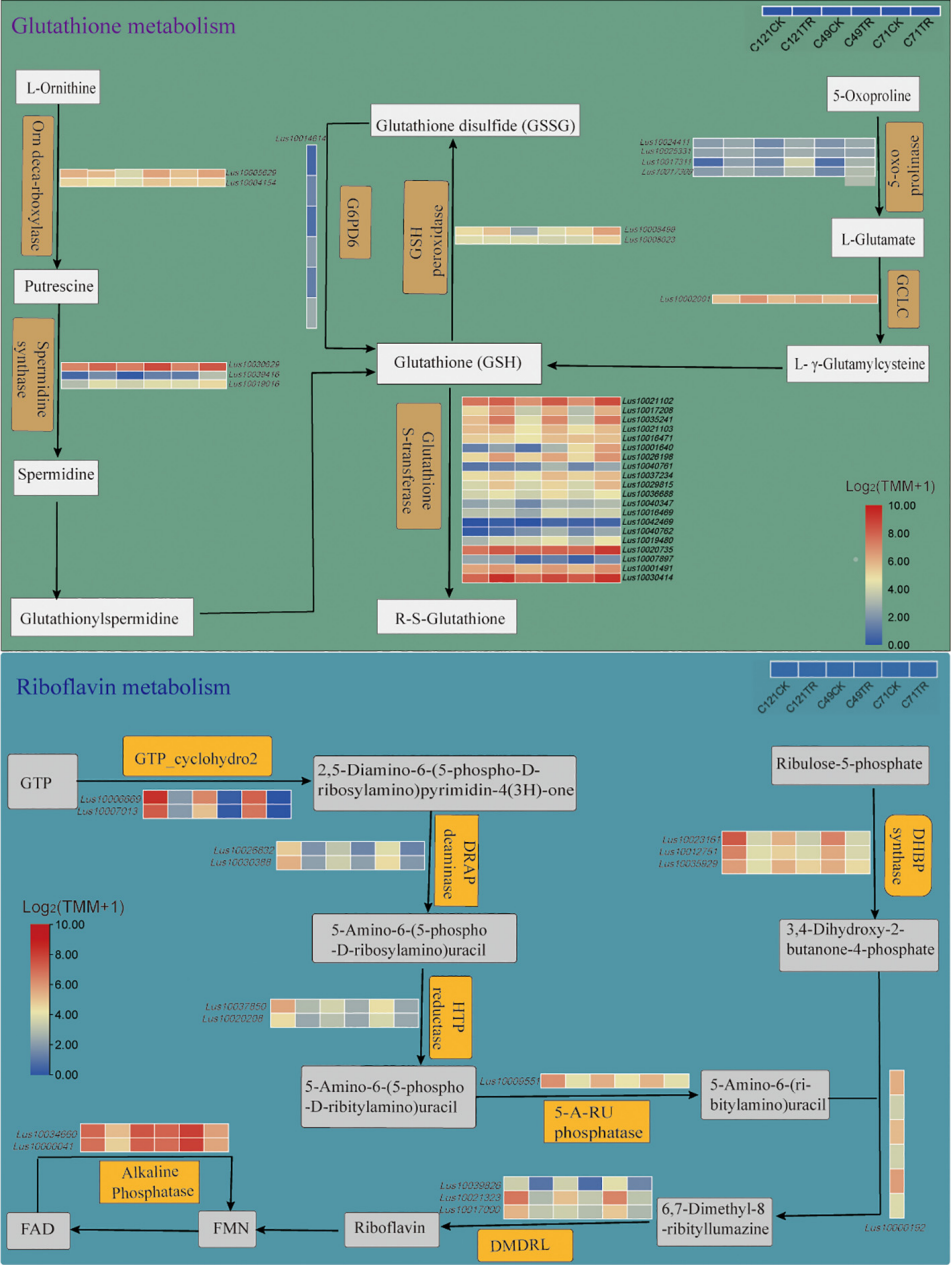
Glutathione metabolism and riboflavin metabolism pathways of flax under salt stress. These pathways were constructed based on the KEGG pathway, in which pathways not involving DEGs were omitted. Refer to **Supplementary Tables S8**, **S9** for DEG information.

Transcriptome analysis identified 21 salt-related DEGs encoding GSTs and 2 DEGs encoding GPXs (**Figure 4**; **Supplementary Table S8**). The genes encoding GSTs were classified into four main groups: Phi, Theta, Tau, and Lambda, all of which displayed upregulated expression under salt stress. It is worth noting that the gene encoding GPX, *Lus10008499*, exhibited significant upregulation in C49 and C71, while its expression levels remained unchanged in C121 under salt stress. Additionally, we observed that the gene *Lus10009135*, which encodes dehydroascorbate reductase (DHAR3) involved in glutathione synthesis, was significantly upregulated only in C121 under salt stress. These observations provide further insights into the involvement of glutathione metabolism in flax’s adaptation to salt stress.

Riboflavin metabolism may be critical to the process of flax salt adaptation (**Figures 2D**, **3D**; **Supplementary Figures S5G–I**, **Supplementary Tables S5**, **S6**). KEGG enrichment pathway analysis identified 16 DEGs in salt-stressed flax root systems, including 2 genes encoding alkaline phosphatase, 4 genes for riboflavin synthase (*LusRibC*), 3 for phosphosynthase (*LusRibB*), 1 gene for phosphatase (*LusPyrP*), 2 for riboflavin-specific deaminase (*LusRibD2* and *LusRibD1*), and 2 GTP cyclohexanase (*LusRibA*) (**Figure 4**; **Supplementary Table S9**). Under salt stress, the expression of all of these genes was notably reduced. However, it is worth noting that among the varieties studied, C121 exhibited a significantly higher expression of five genes encoding RibA and RibB compared to the other varieties. Notably, the gene encoding FAD phosphatase (converts FAD to FMN) showed downregulation in C121 and C71, while no significant change was observed in C49 under salt stress.

### 3.6 Candidate genes of salt tolerance by RNA-seq combined with population-level screening

To further identify candidate genes associated with salt stress, we conducted a subsequent screening using 7,459 salt-related differential genes at the population level in natural flax populations. These genes were mapped into a high-density flax genomic variation map (Guo et al., 2020), resulting in a total of 42,649 SNPs for further analysis. To reveal the degree of contribution of salt-responsive genes to flax salt adaptation, we performed a candidate gene-based association study of relative survival rates in the population. Using the general linear model (GLM) and mixed linear model (MLM), we screened 17 candidate genes associated with survival rates (**Supplementary Figure S7**; **Supplementary Table S10**). Population data indicated that the salt tolerance of oil flax was significantly higher than that of fiber flax (**Supplementary Figure S1E**). Therefore, the allele frequencies of all SNPs within these genes and 2000 bp upstream from the two subgroups were extracted, and π-values were calculated using VCFtools. The nucleotide diversity of its nine genes in the oil flax subgroup was significantly higher than that in the fiber flax subgroup, indicating that some salt-related genes may have undergone positive selection during flax domestication from oil flax to fiber flax (**Supplementary Figure S8**).

**Figure 5:**
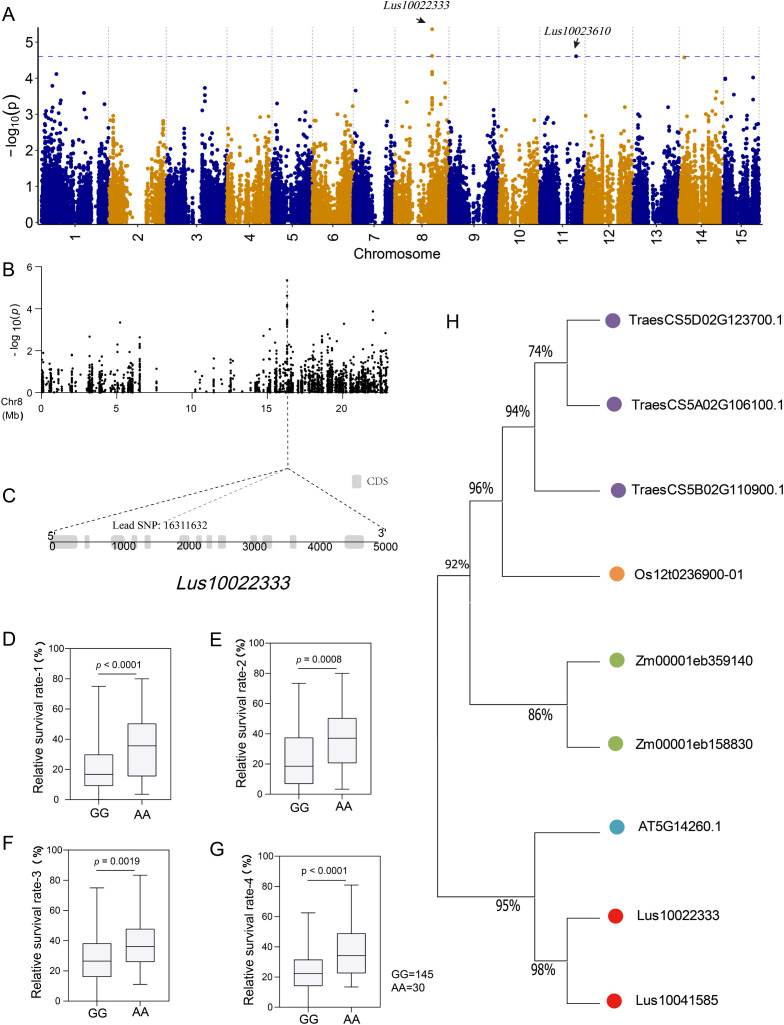
Screening and analyzing genes related to salt response. **(A)** manhattan plot using a general linear model (GLM). The blue line indicates the significance threshold (-log_10_(*P*) = 4.61) using GCE; **(B)** Localized Manhattan map of chromosome 8, the position of the lead SNP (-log_10_ (*P*) = 5.36) is indicated by the dotted line; **(C)** Structure of the gene corresponding to lead SNP; **(D–G)** Box plot for survival rate stratified by genotype at the lead SNP in four environments (repeat 1, repeat 2, repeat 3, and mean 4); **(H)** Phylogenetic tree of *Lus10022333* and its homologs in Arabidopsis, rice, maize, wheat, and flax.

We observed the lead SNP on chromosome 8, which is located in the CDS region of a gene (*Lus10022333*) with a length of 4785 bp (**Figures 5A–C**). There are two major haplotypes based on lead SNP (G/A), and the accessions carrying the AA allele showed a greater survival rate than those with the GG haplotype under salt stress in all four environments (**Figures 5D–G**). To further investigate the function of *Lus10022333*, we conducted a protein blast against a database consisting of Arabidopsis, rice, sunflower, maize, soybean, and flax. The results were filtered based on E-value and identified length, and after removing duplicates, we identified 11 highly homologous proteins (**Figure 5H**). One of the identified proteins in the Arabidopsis database, AT5G14260.1, has been reported to suppress singlet oxygen-induced stress responses by protecting grana margins (Wang et al., 2020). Additionally, Zhang et al. (Zhang et al., 2012) demonstrated that drought stress induces the expression of Rubisco protein methylation-related genes, which play a role in preventing Rubisco protein oxidation and degradation.

**Figure 6:**
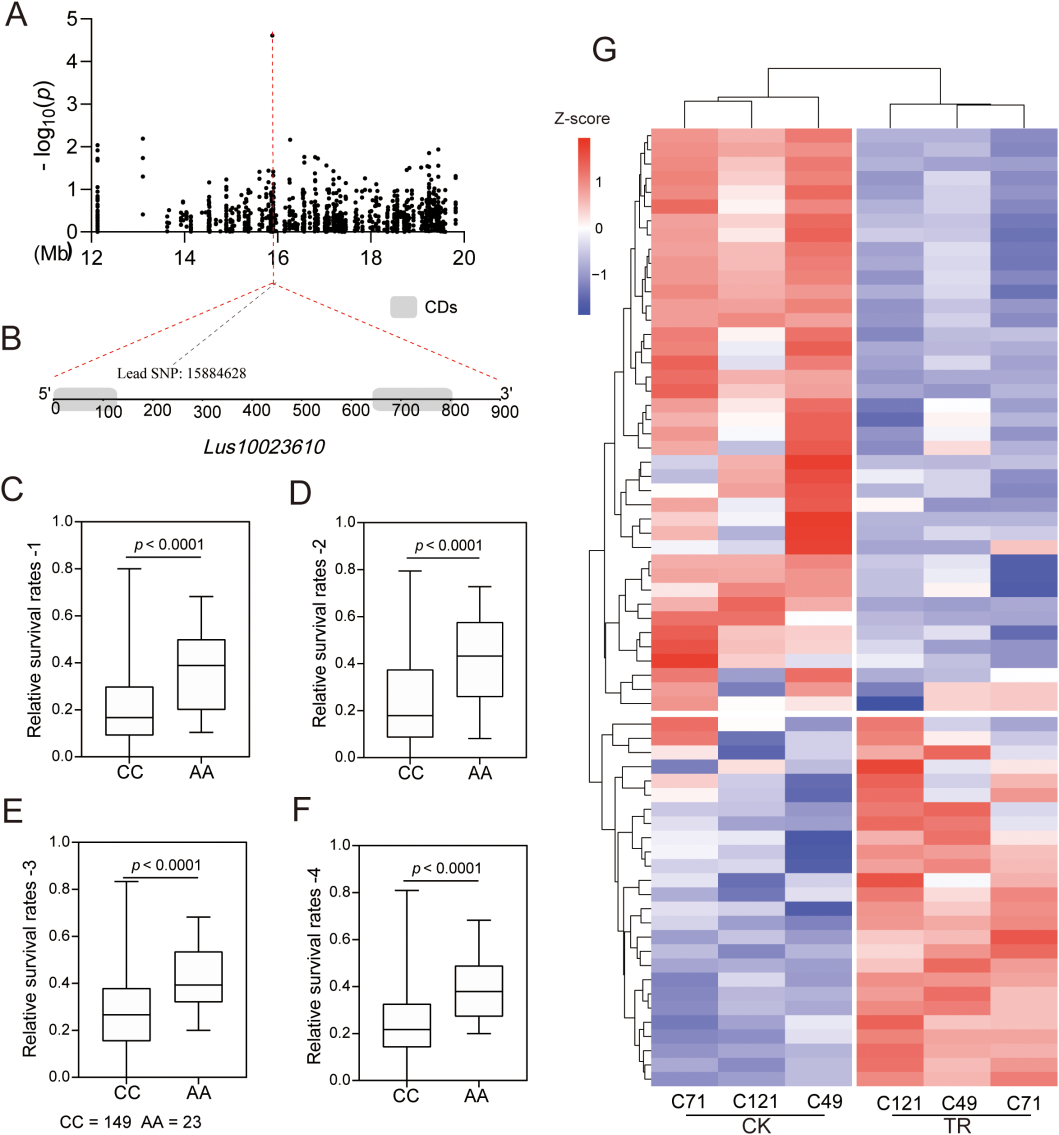
Identification and expression analysis of the bHLH gene. **(A–F)** Identification of a salt-associated core bHLH gene, *Lus10023610*; **(G)** Expression analysis of 67 bHLH genes under salt stress.

Notably, a lead SNP significantly associated with salt stress was located in the interior of a gene encoding a bHLH family transcription factor (*Lus10023610*) (**Figures 6A, B**). Transcriptome analysis showed that the expression of the gene was downregulated in all three varieties under salt stress (**Supplementary Table S4**). Moreover, we identified two major haplotypes based on lead SNP (C/A), and the accessions carrying the AA allele showed a greater survival rate than those with the CC haplotype under salt stress in all four environments (**Figures 6C–F**). These results strongly suggest the involvement of this gene in regulating salt adaptation in flax. Further, we identified a total of 178 genes belonging to the bHLH family in flax, and among them, 67 were responsive to salt stress (**Figure 6G**; **Supplementary Figure S9**; **Supplementary Table S11**). Notably, out of these salt-responsive genes, the expression patterns of 42 differentially expressed genes (DEGs) were similar to the localized gene we focused on. This may indicate a shared motif sequence or a common regulatory pathway among these genes.

## 4 Discussion

Soil salinity is a major environmental stress that restricts the growth and yield of crops (Melino and Tester, 2023). Plant roots have direct contact with the soil and play a critical role in sustaining plant growth and crop yields, particularly in harsh environmental conditions (Galvan-Ampudia and Testerink, 2011). It is necessary to identify salt response and tolerance mechanisms in plant roots to improve crop salt stress resistance. Here, we examine the physiological and transcriptomic changes in the root systems of the three cultivars, and conduct candidate gene mining in conjunction with the population data, providing a comprehensive understanding of the molecular regulation and adaptation of flax to salt stress.

### 4.1 Accumulation of osmotic regulatory substances in response to salt stress

Loss of water, resulting from increased osmotic pressure, presents itself as a significant challenge for plants that strive to thrive in salinized soils (van Zelm et al., 2020). Here, we found that proline and soluble sugar contents were substantially increased in three cultivated flax root systems under salt stress (**Figures 1F, G**). Compatible osmotic substances such as soluble sugars and proline, which increase with salt concentration, are considered to play an important regulatory role in flax’s response to osmotic stress (Li et al., 2022a; Yadav et al., 2022). Meanwhile, transcriptome analysis revealed a large number of salt-induced DEGs that were enriched in starch and sucrose metabolism, as well as amino sugar and nucleotide sugar metabolism (**Figure 2D**; **Supplementary Figures S5G–I**; **Supplementary Table S5**). These genes are involved in regulating the synthesis of osmoregulatory substances. Consistently, Zhao et al. (Wei et al., 2022) also found that the important genes of flax roots regulating salt resistance metabolism under salt stress are involved in regulating sugar metabolism, thus effectively reducing the damage caused by saline-alkali stress by enhancing the osmotic pressure of cells.

Osmoregulatory capacity may be one of the reasons for determining the strength of salt tolerance in flax. The water loss rate of C121 under salt stress was significantly lower than that of the two salt-sensitive varieties, and it exhibited a substantial accumulation of free proline and soluble sugars after salt induction (**Figures 1E–G**). By comparing transcript differences between salt-tolerant and salt-sensitive varieties, we identified some genes that regulate osmotic stress (**Figure 3**; **Supplementary Tables S6**, **S7**). A gene encoding SNRK2E with differential expression between salt-tolerant and salt-sensitive flax varieties (**Supplementary Table S7**), has been demonstrated to play an important role in rice response to osmotic stress (Kobayashi et al., 2004). We also found that five genes encoding water channel proteins (two TIP1:3, two TIP2:2, and one PIP2:2) exhibited significantly higher expression levels in C121 compared to other varieties when subjected to salt stress (**Supplementary Table S7**). Notably, transcriptome investigations and physiological testing revealed that the osmoregulatory capacity of the two salt-sensitive varieties differs significantly as well (**Figures 1F, G**; **Supplementary Figure S10**).

### 4.2 Remodeling ROS homeostasis under salt stress

To cope with the toxicity of ROS, a complete antioxidant system was organized in flax, and the activities of ROS scavengers such as SOD, CAT, and GST were significantly increased under salt stress (Abido and Zsombik, 2019; Li et al., 2022a; Yadav et al., 2022). In physiological experiments, it was observed that three different flax cultivars showed a significant increase in CAT activity when exposed to salt stress (**Figure 1H**). Transcriptome analysis showed that 18 DEGs responsive to salt stress were enriched in the ROS response pathway, and one of them, *LusCYP71B10* (*Lus10030189*), had over tenfold changes across the three flax cultivars under salt stress (**Figure 2D**; **Supplementary Tables S4**, **S5**). Li et al. (Li et al., 2013) revealed that overexpression of *OsCYP71Z2* increased the ability to scavenge ROS in rice. Glutathione metabolism is regarded as one of the crucial antioxidant pathways, and it has been observed that the expression of 21 GST-related genes is significantly induced in response to salt stress (**Figure 4**; **Supplementary Table S8**). Heterologous expression of GST genes from soybean (Jia et al., 2015), oilseed rape (Kao et al., 2016), and tamarisk (Yang et al., 2014) into Arabidopsis can enhance the antioxidant capacity and thus salt tolerance of Arabidopsis. Simultaneously, we have identified a gene, *Lus10025538*, encoding L-ascorbate oxidase that appears to have undergone purifying selection during domestication (**Supplementary Figure S8**). osa-miR12477 can target L-ascorbate oxidase and thereby regulate the oxidative response generated in rice after salt treatment (Dai et al., 2022).

The accumulation and scavenging of ROS may be one of the important reasons for the difference in salt tolerance between the two flax cultivars. The salt-tolerant variety C121 had lower ROS levels than other types during salt stress, according to the results of physiological and biochemical studies (**Figures 1H, I**; **Supplementary Figures S2D, E**). Comparative transcriptome analysis revealed that C121 upregulated more genes related to phenylpropanoid biosynthesis, stilbenoid, diarylheptanoid, and gingerol biosynthesis, and flavonoid synthesis pathways compared to the two salt-sensitive varieties during salt stress (**Figure 3**; **Supplementary Table S6**). Phenylacetones, flavonoids, and terpenoids play a vital role as antioxidants in plants, effectively enhancing their antioxidant capacity through the synthesis of these secondary metabolites (Assaf et al., 2022; Di Ferdinando et al., 2012). Meanwhile, a gene homologous to *AtMYB12*, *Lus10001458*, was identified (**Supplementary Table S7**). This discovery highlights the similar function of *Lus10001458* to *AtMYB12* in enhancing salt and drought tolerance through the elevation of flavonoid and ABA levels in transgenic Arabidopsis (Wang et al., 2016). Interestingly, we found that DHAR3 was upregulated under salt stress only in C121, whereas F4K6Z5 was downregulated under salt stress specifically in salt-sensitive material (**Supplementary Tables S8**, **S10**). However, the junction of these two genes is *LusAPX3*, which is a salt-responsive differentially expressed gene, but no significant differences were observed between salt-tolerant and salt-sensitive materials. It has been shown that APX3 enhances the antioxidant capacity of Arabidopsis and thus responds to salt, drought, and heat stress (Li et al., 2019).

### 4.3 Energy costs affect plant determination to resist adversity

Total energy gain decreases with higher salinity due to reduced photosynthetic rate from induced stomatal closure and damage to cellular and photosynthetic machinery, while stress tolerance mechanisms represent additional costs for the plant to cope with soil salt loads (Munns and Gilliham, 2015). The C121 plants exhibited a relatively dwarf and slow-growing nature, which may account for the notably superior salt tolerance of this cultivar compared to the other two (**Supplementary Figure S2**). Through the application of population genetics, we identified the gene encoding GTP-binding protein beta 1, known as *Lus10042325* (**Supplementary Figures S7**, **S8**; **Supplementary Table S10**). Interestingly, this gene belongs to one of the 497 core genes that are differentially expressed among materials (**Supplementary Table S7**). Heterotrimeric G-proteins play a critical role in regulating various aspects of growth, development, and stress response pathways (Roy Choudhury et al., 2020). Additionally, riboflavin acts as a central component in the cofactors FMN and FAD, essential for a wide array of redox reactions crucial to core energy metabolism (Sa et al., 2016). In the study, it was discovered that certain genes involved in the riboflavin metabolic pathway also showed significant differential expression between salt-tolerant and salt-sensitive materials (**Figure 4**; **Supplementary Table S9**). Notably, comparative transcriptome analysis of the two salt-sensitive materials also revealed the involvement of the riboflavin metabolic pathway (**Supplementary Figure S10**).

Two salt-sensitive flax varieties were identified at the seedling stage: C49, exhibiting green seedlings that rapidly perished under salt stress, and C71, whose leaves wilted and subsequently perished at a slower pace under salt stress (**Supplementary Figure S2A**). Notably, C49 exhibited potential salt tolerance during the germination stage, evidenced by its superior germination rate, root length, and shoot length under salt stress (Li et al., 2022). This disparity in salt tolerance between the germination and seedling stages prompts further consideration regarding salt tolerance mechanisms in flax. A free expenditure of energy affects stress tolerance in plants (Munns and Gilliham, 2015). We compared the transcript information of C49 and C71 and conducted GO and KEGG enrichment analysis on the 856 DEGs. The results of the enrichment analysis indicated that C71 and C49 likely employ different mechanisms when facing salt stress (**Supplementary Figure S10**). During salt stress, the dominant types of gene expression of C71 were involved in catabolic processes like aging, fatty acid metabolism, ascorbate and alternate metabolism, and regulation of the immune system. On the other hand, the significant processes of gene expression in C49 were focused on anabolic processes such as arginine biosynthesis, indole alkaloid biosynthesis, stilbenoid, diarylheptanoid, and gingerol biosynthesis, as well as carbon fixation in photosynthetic organisms. These observations suggest that C49 may require a higher energy supply to harmonize salt resistance and growth.

Photosynthesis is the basis for plant growth and energy supply (Evans, 2013). Puzzlingly, a large number of photosynthesis-related genes were upregulated in the roots of C49 under salt stress (**Supplementary Figures S5E, H**; **Supplementary Table S6**). It is speculated that this may be a defense against adversity. The limitation of photo-assimilates under salinity leads to competition among different physiological processes and organs, with root biomass generally being less affected by excess salinity than aboveground organs (Rewald et al., 2013). Using the genotype data, we correlated the population data with DEG and identified a gene encoding Rubisco methyltransferase, *Lus10022333* (**Figure 5**). Homology matching revealed cognate genes in Arabidopsis and rice with this function to suppress singlet oxygen-induced stress responses by protecting grana margins (Zhang et al., 2012). Accumulation of photosynthesis-related genes in roots under salt stress may represent a strategy of prioritizing root maintenance over above-ground parts, potentially leading to the abandonment of the above-ground tissues.

### 4.4 Transcription factors involved in regulating adversity defense

During plant adaptation to harsh environmental conditions, transcription factors (TFs) play a key role in regulating plant responses to stress (Rui et al., 2023; Wu et al., 2018). In a genome-wide association study, we observed that a lead SNP significantly associated with salt stress was located in the interior of a gene encoding a bHLH family transcription factor (*Lus10023610*) (**Figures 6A, B**). The MYB-bHLH-WD40 protein complex plays a crucial role in regulating secondary metabolism and responding to adverse stress conditions in plants (Pan et al., 2020). Our transcriptome analysis identified 73 MYB, 67 bHLH, and 6 WD40 transcription factors. By consolidating the outcomes of 497 overlapping DEGs from the material combination and 2526 genes responsive to salt stress in C121, we were able to further discern 115 genes (**Supplementary Figures S11A, B**; **Supplementary Table S12**). These genes not only participate in the salt stress response in flax but also potentially contribute to the differences in salt tolerance between salt-tolerant and salt-sensitive cultivars. To gain insights into the function of these genes, we compared them with the genes in Arabidopsis and conducted a protein network interaction analysis (**Supplementary Table S13**). We identified 23 stress-related proteins and three proteins that interacted with the localized gene (**Supplementary Figure S11C**). The upstream 2000 bp sequences of 115 DEGs were extracted for predicting promoter cis-acting elements. Interestingly, a significant number of MYB and G-box elements were identified (**Supplementary Figure S12**). bHLH proteins can bind to specific sequences of the E-box (5’-CANNTG-3’), including the well-studied G-box (5’-CACGTG’) (Pireyre and Burow, 2015). These results provide valuable information for understanding the bHLH transcription factor family in flax.

## 5 Conclusion

In this study, we reported that the salt-tolerant genotype C121 exhibits a unique combination of growth and salt tolerance under salt stress conditions, which has greater osmoregulatory and antioxidant capacity compared to two salt-sensitive varieties, C49 and C71. Comparative root transcriptome analysis showed that C121 induced more genes related to aquaporins, ion transport, and secondary metabolism. Furthermore, we performed a genotype-phenotype association analysis of 7,459 salt-responsive genes and identified nine candidate genes that were selected in domestication. Overall, our study has laid a theoretical groundwork for comprehending the molecular mechanisms behind flax’s response to salt stress, thus holding great significance for selecting and breeding salt-tolerant flax varieties.

## Data Availability

The datasets presented in this study can be found in online repositories. The names of the repository/repositories and accession number(s) can be found below: https://www.ncbi.nlm.nih.gov/, PRJNA1165838 and PRJNA590636.

## References

[B1] Abido W. A. E. Zsombik L. (2019). Effect of salinity on germination characters and seedlings parameters of Egyptian flax cultivars growing in Nyiregyhaza. Acta Ecologica Sin. 39, 102–108. doi: 10.1016/j.chnaes.2018.05.001

[B2] Arif Y. Singh P. Siddiqui H. Bajguz A. Hayat S. (2020). Salinity induced physiological and biochemical changes in plants: An omic approach towards salt stress tolerance. Plant Physiol. Biochem. 156, 64–77. doi: 10.1016/j.plaphy.2020.08.042 32906023

[B3] Ashraf M. Foolad M. R. Tuberosa R. (2013). Crop breeding for salt tolerance in the era of molecular markers and marker-assisted selection. Plant Breed. 132, 10–20. doi: 10.1111/pbr.12000

[B4] Assaf M. Korkmaz A. Karaman Ş. Kulak M. (2022). Effect of plant growth regulators and salt stress on secondary metabolite composition in Lamiaceae species. South Afr. J. Bot. 144, 480–493. doi: 10.1016/j.sajb.2021.10.030

[B5] Atta K. Mondal S. Gorai S. Singh A. P. Kumari A. Ghosh T.. (2023). Impacts of salinity stress on crop plants: improving salt tolerance through genetic and molecular dissection. Front. Plant Sci. 14. doi: 10.3389/fpls.2023.1241736 PMC10540871 37780527

[B6] Bradbury P. J. Zhang Z. Kroon D. E. Casstevens T. M. Ramdoss Y. Buckler E. S. (2007). TASSEL: software for association mapping of complex traits in diverse samples. Bioinformatics 23, 2633–2635. doi: 10.1093/bioinformatics/btm308 17586829

[B7] Chen L. Meng Y. Yang W. LV Q. Zhou L. Liu S.. (2023). Genome-wide analysis and identification of TaRING-H2 gene family and TaSDIR1 positively regulates salt stress tolerance in wheat. Int. J. Biol. Macromolecules. 242, 125162. doi: 10.1016/j.ijbiomac.2023.125162 37263334

[B8] Corwin D. L. (2020). Climate change impacts on soil salinity in agricultural areas. Eur. J. Soil Sci. 72, 842–862. doi: 10.1111/ejss.13010

[B9] Dai L. Li P. Li Q. Leng Y. Zeng D. Qian Q. (2022). Integrated multi-omics perspective to strengthen the understanding of salt tolerance in rice. Int. J. Mol. Sci. 23. doi: 10.3390/ijms23095236 PMC9105537 35563627

[B10] Di Ferdinando M. Brunetti C. Fini A. Tattini M. (2012). “Flavonoids as antioxidants in plants under abiotic stresses,” in Abiotic Stress Responses in Plants, 159–179.

[B11] Du F. Wang Y. Wang J. Li Y. Zhang Y. Zhao X.. (2023). The basic helix-loop-helix transcription factor gene. OsbHLH38 plays key role controlling Rice salt tolerance. J. Integr. Plant Biol. 65, 1859–1873. doi: 10.1111/jipb.13489 36988217

[B12] El Mamoun I. Bouzroud S. Zouine M. Smouni A. (2023). The knockdown of *AUXIN RESPONSE FACTOR 2* confers enhanced tolerance to salt and drought stresses in tomato (*Solanum lycopersicum* L.). Plants 12, 1–23. doi: 10.3390/plants12152804 PMC10420960 37570958

[B13] Evans J. R. (2013). Improving photosynthesis. Plant Physiol. 162, 1780–1793. doi: 10.1104/pp.113.219006 23812345 PMC3729760

[B14] Galvan-Ampudia C. S. Testerink C. (2011). Salt stress signals shape the plant root. Curr. Opin. Plant Biol. 14, 296–302. doi: 10.1016/j.pbi.2011.03.019 21511515

[B15] Guo D. Jiang H. Yan W. Yang L. Ye J. Wang Y.. (2020). Resequencing 200 flax cultivated accessions identifies candidate genes related to seed size and weight and reveals signatures of artificial selection. Front. Plant Sci. 10. doi: 10.3389/fpls.2019.01682 PMC6976528 32010166

[B16] Hall L. M. Booker H. Siloto R. M. Jhala A. J. Weselake R. J. (2016). “Flax (*Linum usitatissimum* L.),” in Industrial oil crops (AOCS Press), 157–194.

[B17] Jia B. Sun M. Sun X. Li R. Wang Z. Wu J.. (2015). Overexpression of GsGSTU13 and *SCMRP* in Medicago sativa confers increased salt–alkaline tolerance and methionine content. Physiologia plantarum 156, 176–189. doi: 10.1111/ppl.12350 26010993

[B18] Jiang C. Johkan M. Hohjo M. Tsukagoshi S. Maruo T. (2017). A correlation analysis on chlorophyll content and SPAD value in tomato leaves. HortResearch 71, 37–42. doi: 10.20776/S18808824-71-P37

[B19] Jiang Z. Zhou X. Tao M. Yuan F. Liu L. Wu F.. (2019). Plant cell-surface GIPC sphingolipids sense salt to trigger Ca^2+^ influx. Nature 572, 341–346. doi: 10.1038/s41586-019-1449-z 31367039

[B20] Kao C.-W. Bakshi M. Sherameti I. Dong S. Reichelt M. Oelmüller R.. (2016). A Chinese cabbage (*Brassica campetris* subsp. Chinensis) τ-type glutathione-S-transferase stimulates Arabidopsis development and primes against abiotic and biotic stress. Plant Mol. Biol. 92, 643–659. doi: 10.1007/s11103-016-0531-2 27796720

[B21] Kobayashi Y. Yamamoto S. Minami H. Kagaya Y. Hattori T. (2004). Differential activation of the rice sucrose nonfermenting1–related protein kinase2 family by hyperosmotic stress and abscisic acid[W. Plant Cell 16, 1163–1177. doi: 10.1105/tpc.019943 15084714 PMC423207

[B22] Li X. Guo D. Xue M. Li G. Yan Q. Jiang H.. (2022). Genome-wide association study of salt tolerance at the seed germination stage in flax (*Linum usitatissimum* L.). Genes 13, 1–13. doi: 10.3390/genes13030486 PMC8949523 35328040

[B23] Li Y. Chen J. Li X. Jiang H. Guo D. Xie F.. (2022a). Adaptive response and transcriptomic analysis of flax (*Linum usitatissimum* L.) seedlings to salt stress. Genes 13, 1–15. doi: 10.3390/genes13101904 PMC9601370 36292789

[B24] Li Y. Jiang H. Xie L. (2022b). Review of plant adaptation mechanism to salt stress. J. Plant Genet. Resour. 23, 1585–1593. doi: 10.13430/j.cnki.jpgr.20220518003

[B25] Li C. Kong J.-R. Yu J. He Y.-Q. Yang Z.-K. Zhuang J.-J.. (2023). DNA demethylase gene OsDML4 controls salt tolerance by regulating the ROS homeostasis and the JA signaling in rice. Environ. Exp. Bot. 209, 1–11. doi: 10.1016/j.envexpbot.2023.105276

[B26] Li Z. Li J. Bing J. Zhang G. (2019). The role analysis of APX gene family in the growth and developmental processes and in response to abiotic stresses in *Arabidopsis thaliana* . Hereditas (Beijing) 41, 534–547. doi: 10.16288/j.yczz.19-026 31257201

[B27] Li W. Shao M. Yang J. Zhong W. Okada K. Yamane H.. (2013). Oscyp71Z2 involves diterpenoid phytoalexin biosynthesis that contributes to bacterial blight resistance in rice. Plant Sci. 207, 98–107. doi: 10.1016/j.plantsci.2013.02.005 23602104

[B28] Li M.-X. Yeung J. M. Y. Cherny S. S. Sham P. C. (2011). Evaluating the effective numbers of independent tests and significant p-value thresholds in commercial genotyping arrays and public imputation reference datasets. Hum. Genet. 131, 747–756. doi: 10.1007/s00439-011-1118-2 22143225 PMC3325408

[B29] Li J. L. Zhong L. L. Wang J. Ma T. Mao K. S. Zhang L. (2020). Genomic insights into speciation history and local adaptation of an alpine aspen in the Qinghai–Tibet Plateau and adjacent highlands. J. Systematics Evol. 59, 1220–1231. doi: 10.1111/jse.12665

[B30] Liao Y. Smyth G. K. Shi W. (2014). featureCounts: an efficient general purpose program for assigning sequence reads to genomic features. Bioinformatics 30, 923–930. doi: 10.1093/bioinformatics/btt656 24227677

[B31] Love M. I. Huber W. Anders S. (2014). Moderated estimation of fold change and dispersion for RNA-seq data with DESeq2. Genome Biol. 15, 550. doi: 10.1186/s13059-014-0550-8 25516281 PMC4302049

[B32] Lu P. Dai S.-Y. Yong L.-T. Zhou B.-H. Wang N. Dong Y.-Y.. (2023). A soybean sucrose non-fermenting protein kinase 1 gene, GmSNF1, positively regulates plant response to salt and salt–alkali stress in transgenic plants. Int. J. Mol. Sci. 24, 1–16. doi: 10.3390/ijms241512482 PMC10419833 37569858

[B33] Mao T. Geng Z. Zhang Y. Xue W. Ma L. Yang J.. (2023). Genome-wide characterization of NmrA-like proteins and the regulatory function of soybean GmNmrA6 in response to salt and oxidative stresses. Environ. Exp. Bot. 213, 105447–105459. doi: 10.1016/j.envexpbot.2023.105447

[B34] Mekawy A. M. M. Assaha D. V. M. Ueda A. (2019). Differential salt sensitivity of two flax cultivars coincides with differential sodium accumulation, biosynthesis of osmolytes and antioxidant enzyme activities. J. Plant Growth Regul. 39, 1119–1126. doi: 10.1007/s00344-019-10048-5

[B35] Melino V. Tester M. (2023). Salt-tolerant crops: time to deliver. Annu. Rev. Plant Biol. 74, 671–696. doi: 10.1146/annurev-arplant-061422-104322 36854479

[B36] Munns R. Gilliham M. (2015). Salinity tolerance of crops – what is the cost? New Phytol. 208, 668–673. doi: 10.1111/nph.13519 26108441

[B37] Pan J. Li Z. Dai S. Ding H. Wang Q. Li X.. (2020). Integrative analyses of transcriptomics and metabolomics upon seed germination of foxtail millet in response to salinity. Sci. Rep. 10, 13660. doi: 10.1038/s41598-020-70520-1 32788682 PMC7423953

[B38] Pireyre M. Burow M. (2015). Regulation of MYB and bHLH Transcription Factors: A Glance at the Protein Level. Mol. Plant 8, 378–388. doi: 10.1016/j.molp.2014.11.022 25667003

[B39] Qin H. Wang J. Chen X. Wang F. Peng P. Zhou Y.. (2019). Rice OsDOF15 contributes to ethylene-inhibited primary root elongation under salt stress. New Phytol. 223, 798–813. doi: 10.1111/nph.15824 30924949

[B40] Rewald B. Shelef O. Ephrath J. E. Rachmilevitch S. (2013). “Adaptive plasticity of salt-stressed root systems,” in Ecophysiology and responses of plants under salt stress, 169–201.

[B41] Roy Choudhury S. Li M. Lee V. Nandety R. S. Mysore K. S. Pandey S. (2020). Flexible functional interactions between G-protein subunits contribute to the specificity of plant responses. Plant J. 102, 207–221. doi: 10.1111/tpj.14714 32034949

[B42] Rui Z. Pan W. Zhao Q. Hu H. Li X. Xing L.. (2023). Genome-wide identification, evolution and expression analysis of NAC gene family under salt stress in wild emmer wheat (Triticum dicoccoides. L). Int. J. Biol. Macromolecules. 230, 123376. doi: 10.1016/j.ijbiomac.2023.123376 36709820

[B43] Sa N. Rawat R. Thornburg C. Walker K. D. Roje S. (2016). Identification and characterization of the missing phosphatase on the riboflavin biosynthesis pathway in *Arabidopsis thaliana* . Plant J. 88, 705–716. doi: 10.1111/tpj.13291 27490826

[B44] Shen X. Wang Y. Zhang Y. Guo W. Jiao Y. Zhou X. (2018). Overexpression of the wild soybean R2R3-MYB transcription factor GsMYB15 enhances resistance to salt stress and *Helicoverpa armigera* in transgenic Arabidopsis. Int. J. Mol. Sci. 19, 3958. doi: 10.3390/ijms19123958 30544851 PMC6321161

[B45] van Zelm E. Zhang Y. Testerink C. (2020). Salt tolerance mechanisms of plants. Annu. Rev. Plant Biol. 71, 403–433. doi: 10.1146/annurev-arplant-050718-100005 32167791

[B46] Wagner G. P. Kin K. Lynch V. J. (2012). Measurement of mRNA abundance using RNA-seq data: RPKM measure is inconsistent among samples. Theory Biosci. 131, 281–285. doi: 10.1007/s12064-012-0162-3 22872506

[B47] Wang F. Kong W. Wong G. Fu L. Peng R. Li Z.. (2016). AtMYB12 regulates flavonoids accumulation and abiotic stress tolerance in transgenic Arabidopsis thaliana. Mol. Genet. Genomics 291, 1545–1559. doi: 10.1007/s00438-016-1203-2 27033553

[B48] Wang L. Leister D. Guan L. Zheng Y. Schneider K. Lehmann M.. (2020). The Arabidopsis SAFEGUARD1 suppresses singlet oxygen-induced stress responses by protecting grana margins. Proc. Natl. Acad. Sci. 117, 6918–6927. doi: 10.1073/pnas.1918640117 32161131 PMC7104384

[B49] Wang N. Lin Y. Qi F. Xiaoyang C. Peng Z. Yu Y.. (2022). Comprehensive analysis of differentially expressed genes and epigenetic modification-related expression variation induced by saline stress at seedling stage in fiber and oil flax, *Linum usitatissimum* L. Plants 11, 2053. doi: 10.3390/plants11152053 35956530 PMC9370232

[B50] Wei Z. Li Z. Jianping Z. Yanni Q. Liming W. Yaping X.. (2022). Conjoint transcriptome and metabolome analysis of the response mechanism of flax root to salt stress. Pratacultural Sci. 39, 1151–1164. doi: 10.11829/j.issn.1001-0629.2021-0529

[B51] Wu J. Zhao Q. Sun D. Wu G. Zhang L. Yuan H.. (2018). Transcriptome analysis of flax (*Linum usitatissimum* L.) undergoing osmotic stress. Ind. Crops Products 116, 215–223. doi: 10.1016/j.indcrop.2018.02.035

[B52] Wu J. Zhao Q. Wu G. Yuan H. Ma Y. Lin H.. (2019). Comprehensive analysis of differentially expressed unigenes under NaCl stress in flax (*Linum usitatissimum* L.) using RNA-Seq. Int. J. Mol. Sci. 20, 369–283. doi: 10.3390/ijms20020369 30654562 PMC6359340

[B53] Yadav B. Kaur V. Narayan O. P. Yadav S. K. Kumar A. Wankhede D. P. (2022). Integrated omics approaches for flax improvement under abiotic and biotic stress: Current status and future prospects. Front. Plant Sci. 13. doi: 10.3389/fpls.2022.931275 PMC9358615 35958216

[B54] Yang Y. Guo Y. (2018). Unraveling salt stress signaling in plants. J. Integr. Plant Biol. 60, 796–804. doi: 10.1111/jipb.12689 29905393

[B55] Yang G. Wang Y. Xia D. Gao C. Wang C. Yang C. (2014). Overexpression of a GST gene (ThGSTZ1) from Tamarix hispida improves drought and salinity tolerance by enhancing the ability to scavenge reactive oxygen species. Plant Cell Tissue Organ Culture (PCTOC) 117, 99–112. doi: 10.1007/s11240-014-0424-5

[B56] Yu Y. Huang W. Chen H. Wu G. Yuan H. Song X.. (2014). Identification of differentially expressed genes in flax (*Linum usitatissimum* L.) under saline–alkaline stress by digital gene expression. Gene 549, 113–122. doi: 10.1016/j.gene.2014.07.053 25058012

[B57] Yu Y. Wang J. Li S. Kakan X. Zhou Y. Miao Y.. (2019). Ascorbic acid integrates the antagonistic modulation of ethylene and abscisic acid in the accumulation of reactive oxygen species. Plant Physiol. 179, 1861–1875. doi: 10.1104/pp.18.01250 30723177 PMC6446745

[B58] Yu J. Zhu C. Xuan W. An H. Tian Y. Wang B.. (2023). Genome-wide association studies identify OsWRKY53 as a key regulator of salt tolerance in rice. Nat. Commun. 14, 1–12. doi: 10.1038/s41467-023-39167-0 37321989 PMC10272163

[B59] Zhang H. Yu F. Xie P. Sun S. Qiao X. Tang S.. (2023). A Gγ protein regulates alkaline sensitivity in crops. Science 379, 1–14. doi: 10.1126/science.ade8416 36952416

[B60] Zhang X. Zhou J. Han Z. Shang Q. Wang Z. Gu X.. (2012). Active methyl cycle and transfer related Gene expression in response to drought stress in rice leaves. Rice Sci. 19, 86–93. doi: 10.1016/s1672-6308(12)60026-2

[B61] Zhou Y.-B. Liu C. Tang D.-Y. Yan L. Wang D. Yang Y.-Z.. (2018). The receptor-Like cytoplasmic Kinase STRK1 phosphorylates and activates CatC, thereby regulating H_2_O_2_ homeostasis and improving salt tolerance in rice. Plant Cell 30, 1100–1118. doi: 10.1105/tpc.17.01000 29581216 PMC6002193

