# Supplementary material for: Integrating RNA-seq and population genomics to elucidate salt tolerance mechanisms in flax (Linum usitatissimum L.)
Source: Front Plant Sci. 2024 Nov 19;15:1442286. doi: 10.3389/fpls.2024.1442286 (PMC11616478; doi:10.3389/fpls.2024.1442286)
Supplement: Supplementary file 1 [file Table1.docx]

Supplementary Figures





**Supplementary Figure 1.** A comprehensive evaluation of salt tolerance of 200 flax germplasm at seedling stage. (A) Screening population salt concentration; (B) Phenotypic differences in survival rate under salt stress at seedling stage; (C) Correlation of survival rate between environmental repetitions. (D) Statistics on the relative survival of 200 flax materials; (E) Distribution of survival rate in subpopulations. RS represents relative survival, 4 represents the average of three experiments, and 1, 2, and 3 represent the experiment names, respectively.


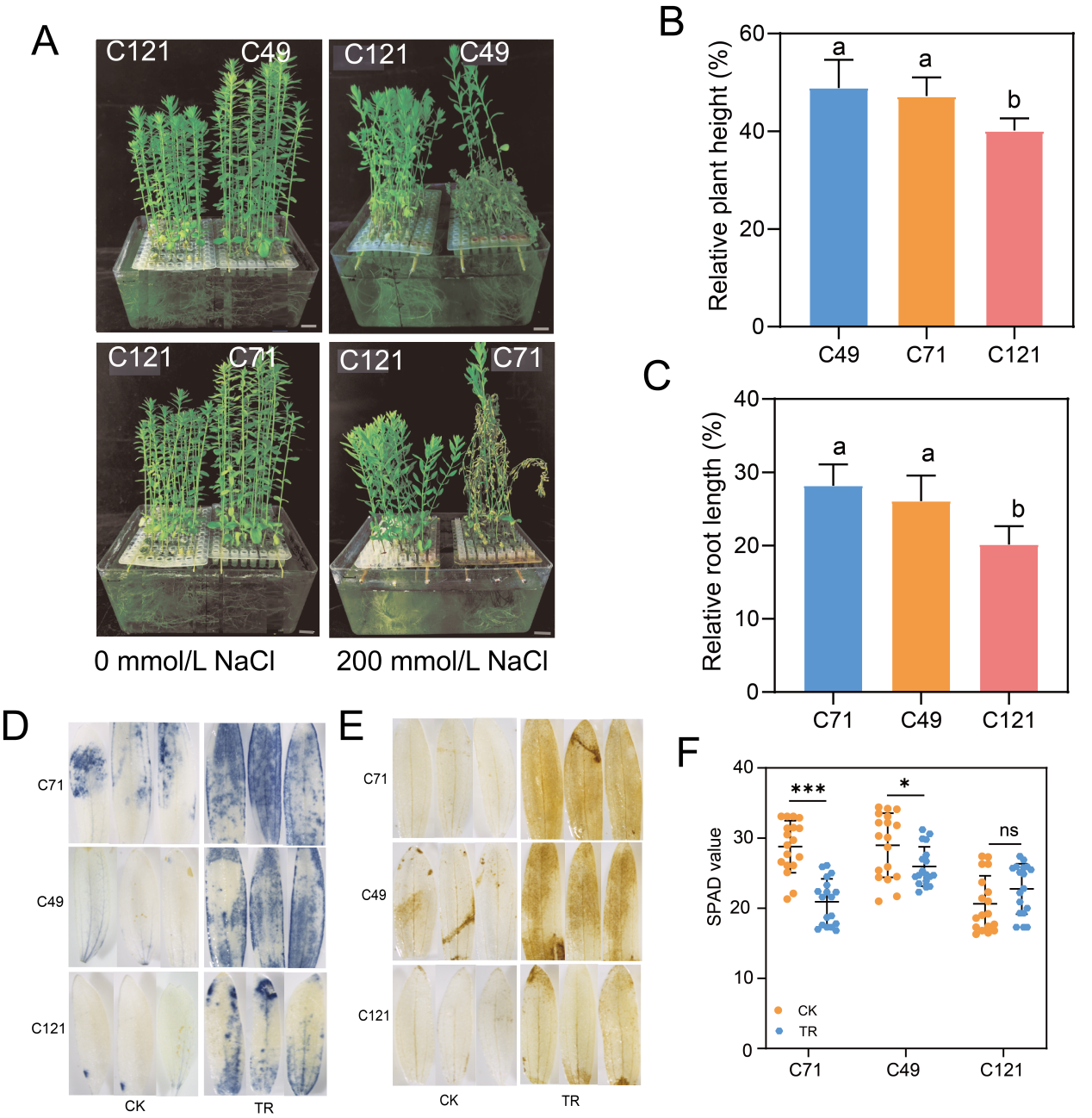


**Supplementary Figure 2.** Statistical changes in the growth of flax. (A) Appearance of flax seedlings treated for 7 days (scale bar = 1 cm); (B) Relative plant height and (C) relative root length under normal concentrations. Seedlings were tested before and after five days of treatment. Error bars represent mean ± SD (n > 3), MRT, adjusted *P* < 0.001 is indicated by different lowercase letters; (D) and (E) NBT and DAB staining of leaves after 3 days of salt treatment, respectively; (F) Leaf SPAD values after 3 days of salt treatment; Independent samples t-test, ns without significant differences, * *P* < 0.05, *** *P* < 0.001


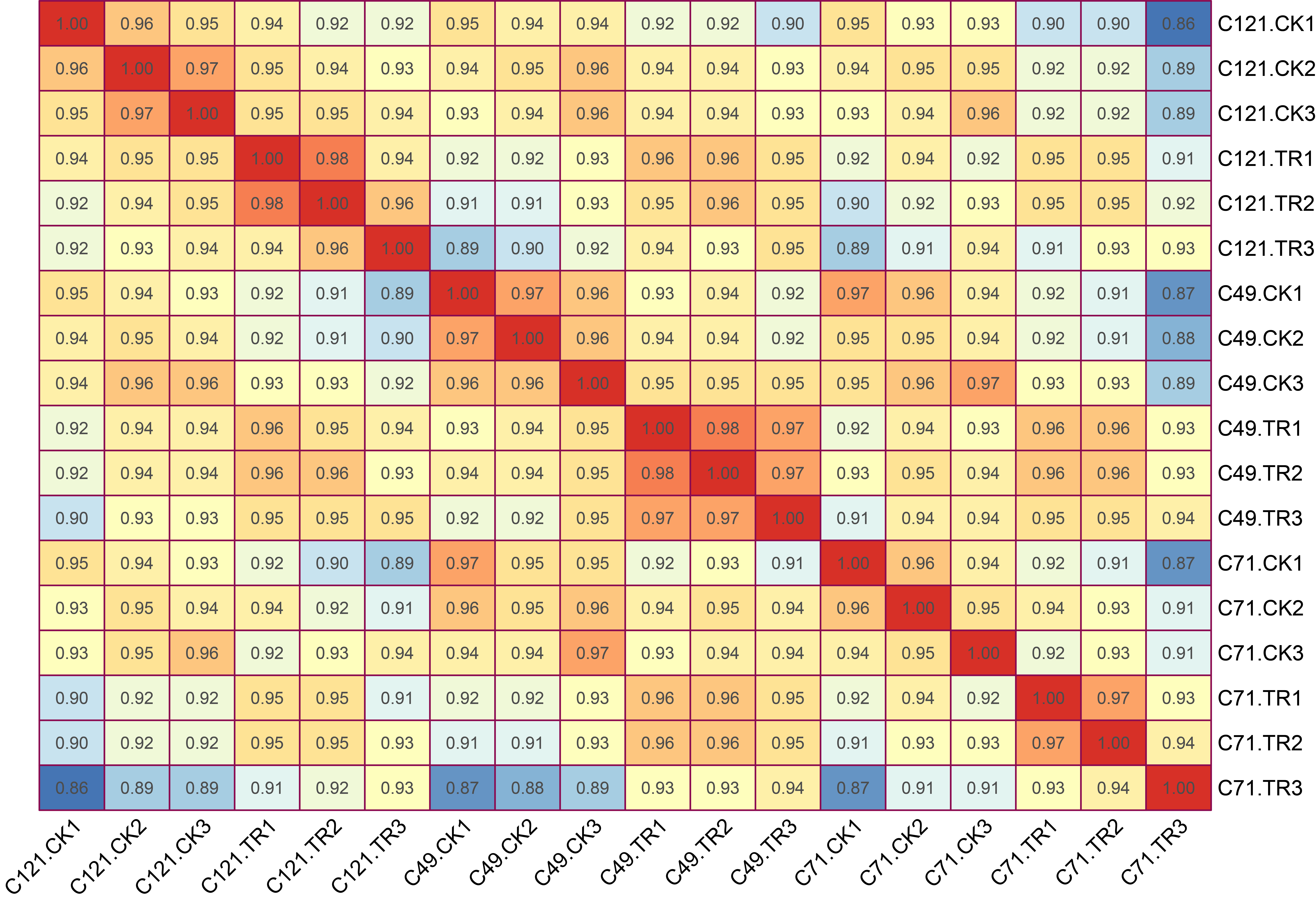


**Supplementary Figure 3.** Correlation of transcriptome samples


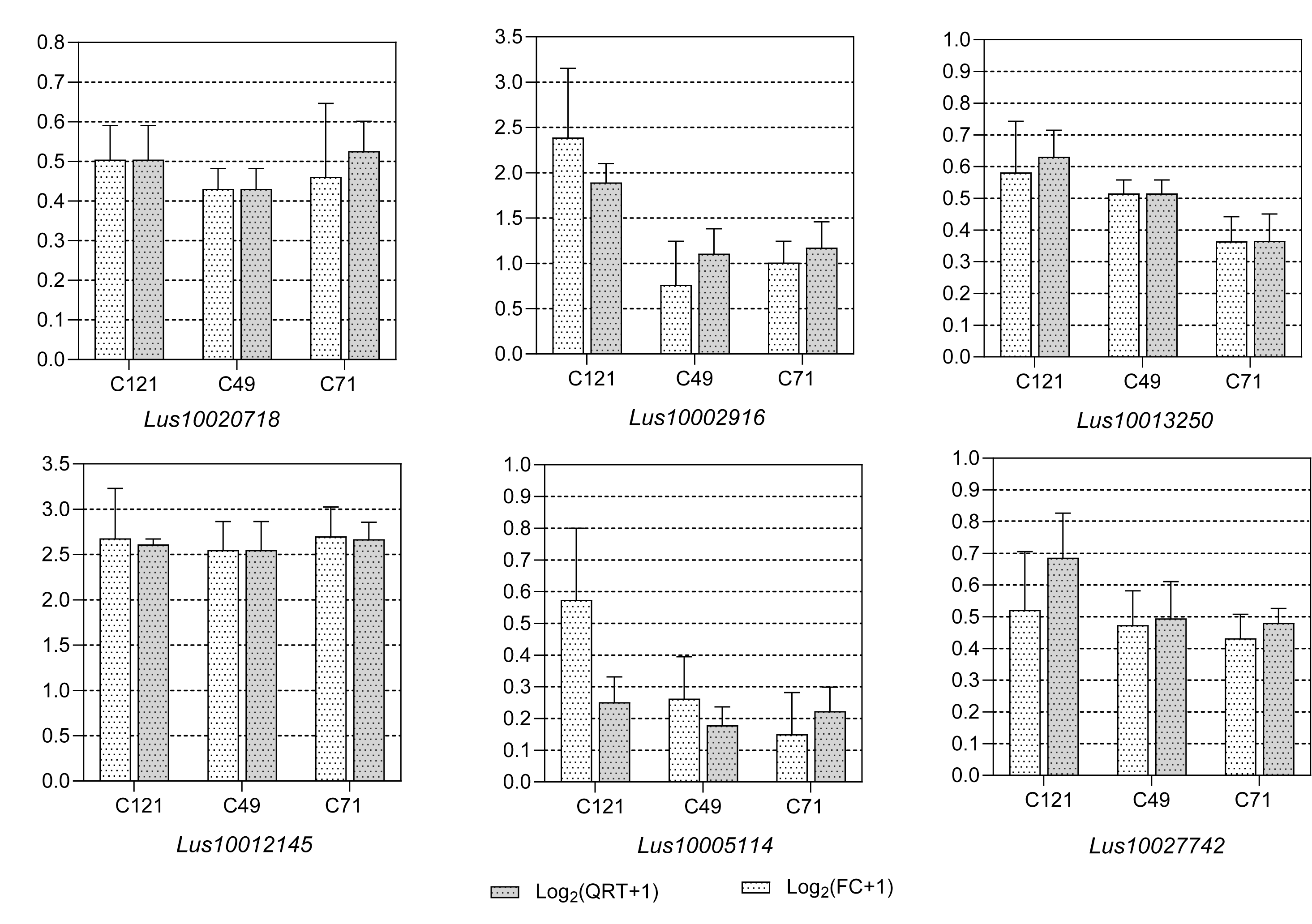


**Supplementary Figure 4.** Validation of the RNA-seq data expression profile with qRT-PCR


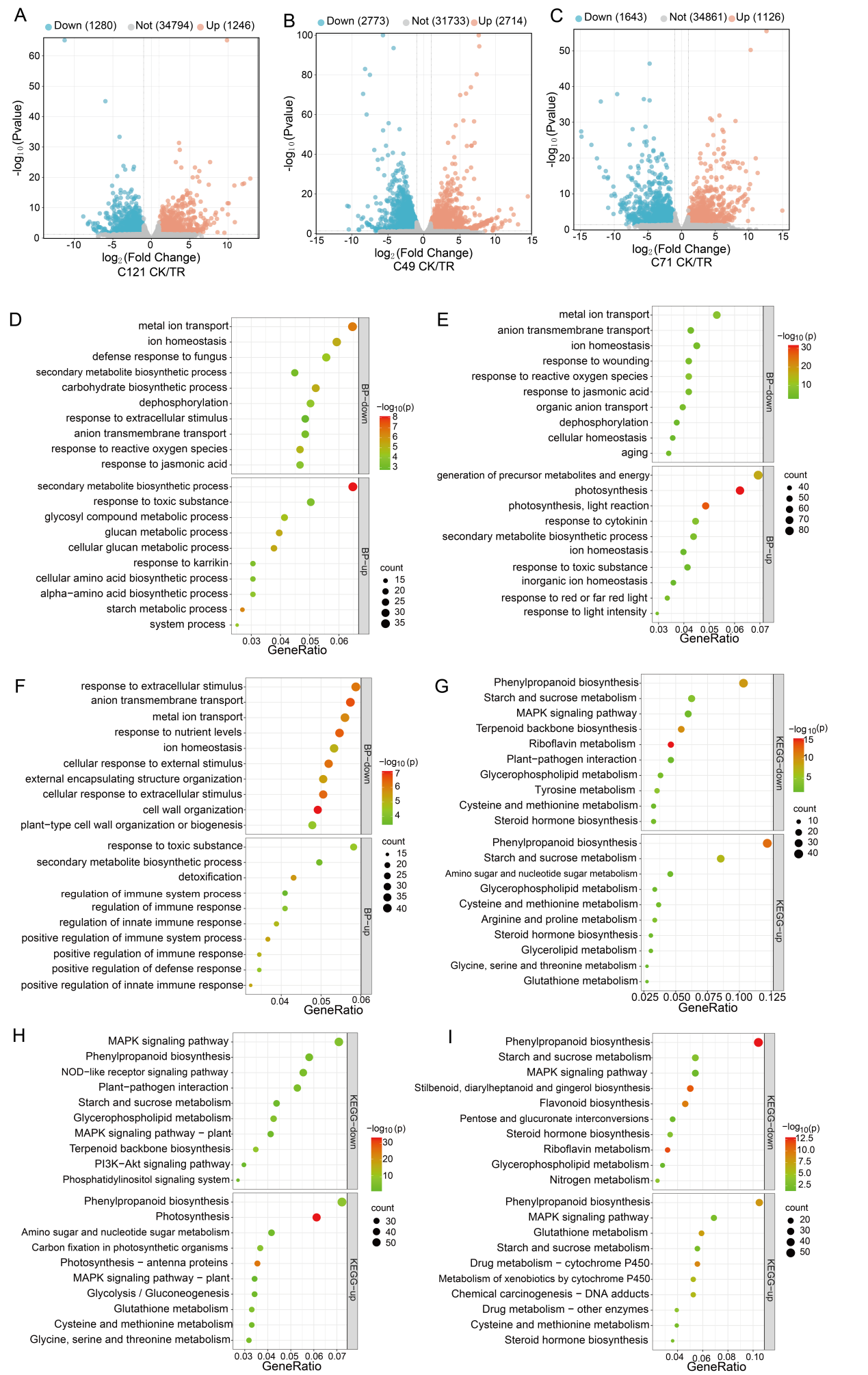


**Supplementary** **Figure 5.** Screening and enrichment analysis of DEGs in three materials under salt stress. Volcano plots based on salt-responsive DEGs in C121 (A), C49 (B), and C71 (C); (D-I) GO and KEGG enrichment analysis plots for C121, C49, and C71, respectively. The enrichment plots were ranked from top to bottom by count number, taking the top 10 upregulated and downregulated terms.


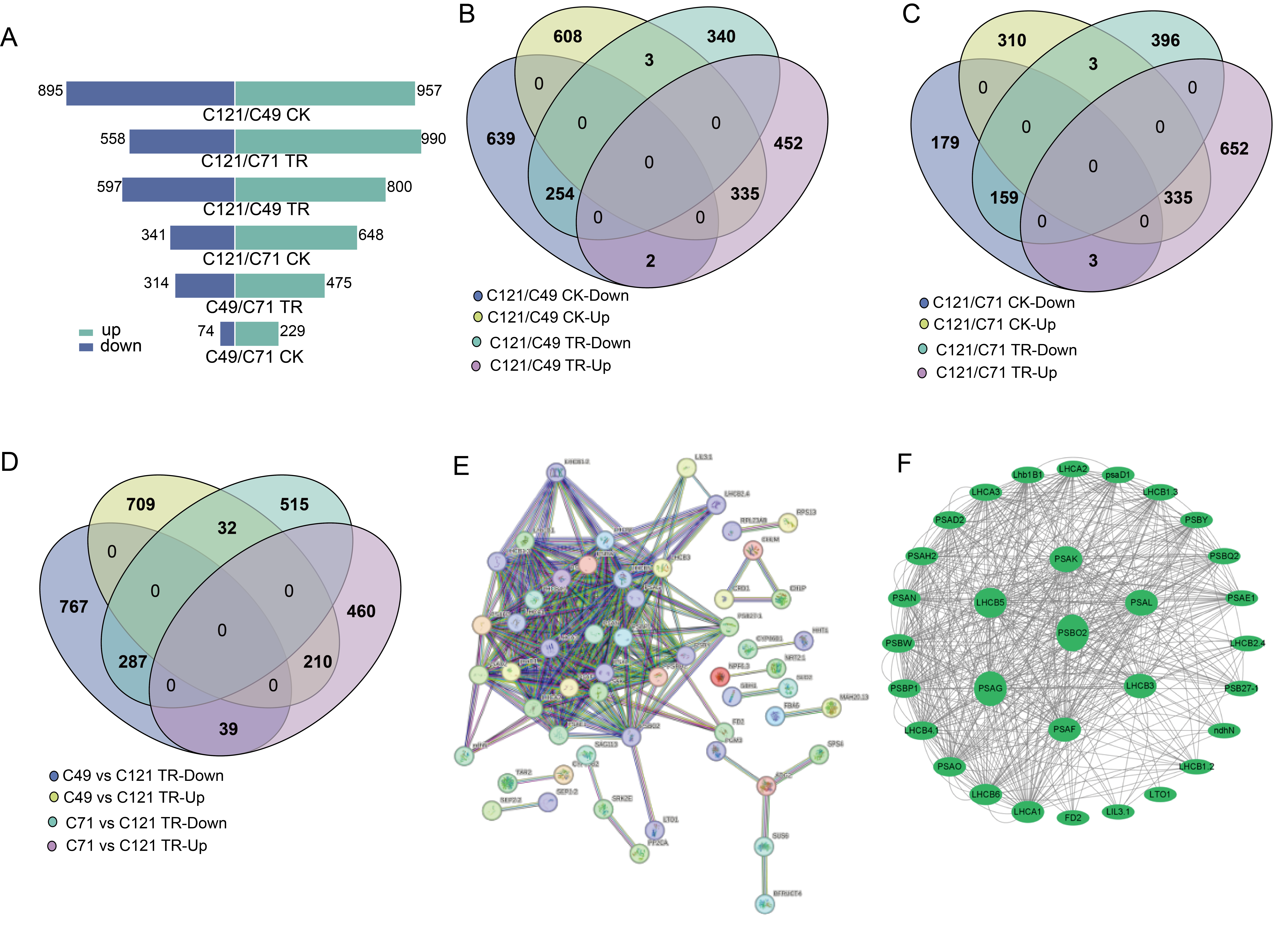


**Supplemental** **Figure 6.** Differential expression gene screen based on regulatory differences between salt-resistant and salt-sensitive materials





**Supplemental Figure 7.** Population-level screening for salt-related core DEGs using a natural population containing 200 flax accessions. GLM and MLM were utilized to associate relative survival rates of repeat 1(A, B), repeat 2(C, D), repeat 3(E, F), and mean 4(G, H) with salt-responsive genes, respectively. The blue dotted line indicates the significance threshold (-log_10_ (*P*) = 4)


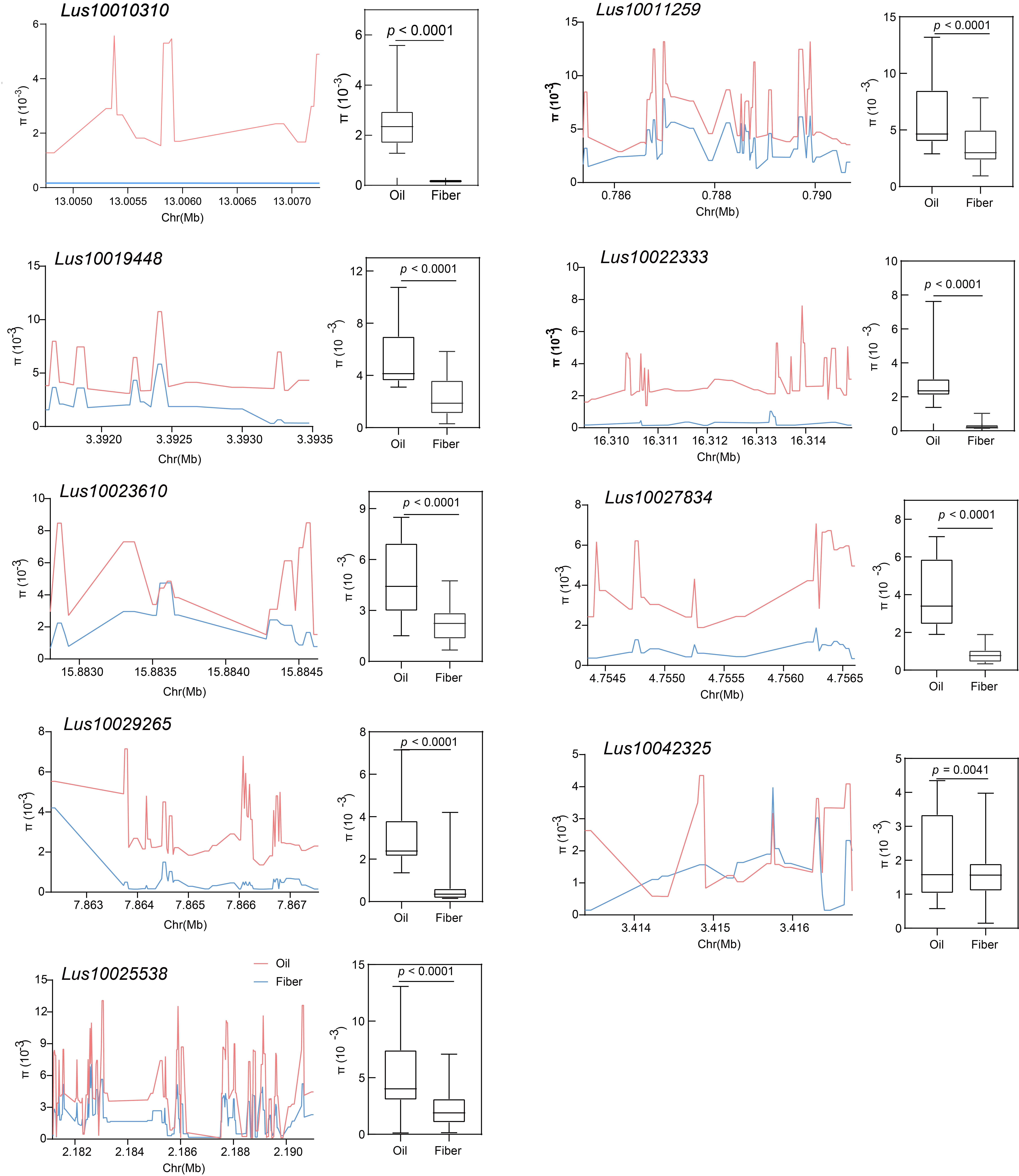


**Supplemental Figure 8.** Statistics of *π*-values from 9 candidate genes and their upstream 2000bp





**Supplementary Figure 9.** Phylogenetic tree, gene expression, and motif analysis of the 67 bHLH genes


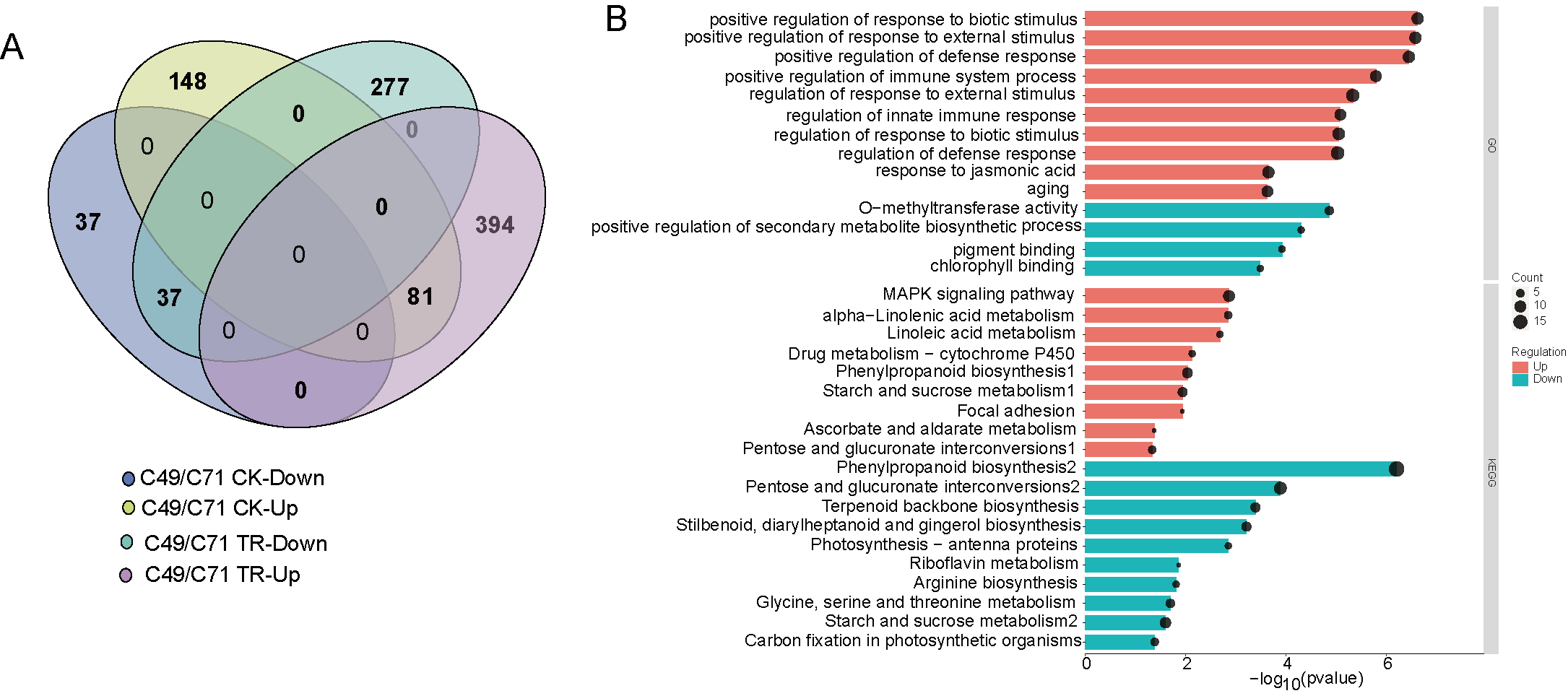


**Supplementary Figure 10.** Genetic differences of salt-sensitive materials to salt stress. (A) Venn diagram of DEGs between two sensitive materials and (B) dotted line graph based on TOP20 terms from GO and KEGG enrichment


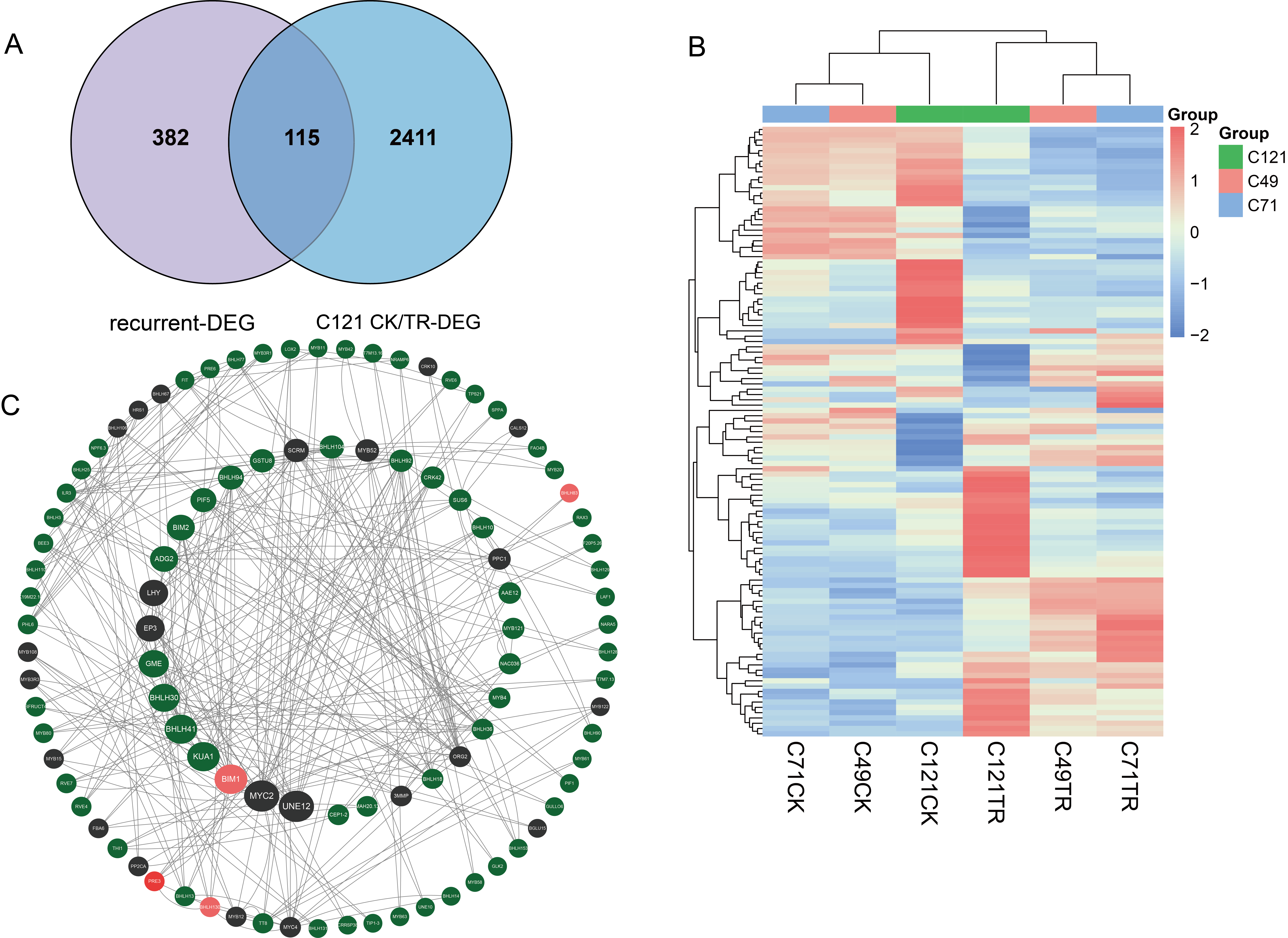


**Supplementary Figure 11.** Interaction prediction of transcription factors (A) Wayne plots between DEGs common to the resistant and two sensitive materials and DEGs under salt stress in the resistant material; (B) Expression analysis of 115 DGEs; (C) Interaction analysis between 115 DEGS and DEGs of bHLH, MYB, WD40 under salt stress. Black represents a proven association with adversity stress in Arabidopsis, and red represents interactions with the localized genes (*Lus10023610*).





**Supplementary Figure 12.** Promoter cis-acting element predictions for 115 core DEGs (PlantCare: http://bioinformatics.psb.ugent.be/webtools/plantcare/html/)
